# The Effect of Mutations on Drug Sensitivity and Kinase Activity of Fibroblast Growth Factor Receptors: A Combined Experimental and Theoretical Study

**DOI:** 10.1016/j.ebiom.2015.02.009

**Published:** 2015-02-15

**Authors:** Tom D. Bunney, Shunzhou Wan, Nethaji Thiyagarajan, Ludovico Sutto, Sarah V. Williams, Paul Ashford, Hans Koss, Margaret A. Knowles, Francesco L. Gervasio, Peter V. Coveney, Matilda Katan

**Affiliations:** a Institute of Structural and Molecular Biology, Division of Biosciences, University College London, Gower St., London WC1E 6BT, UK; b Centre for Computational Science, Department of Chemistry, University College London, 20 Gordon St., London WC1H 0AJ, UK; c Institute of Structural and Molecular Biology, Department of Chemistry, University College London, Gower St., London WC1E 6BT, UK; d Section of Experimental Oncology, Leeds Institute of Molecular Medicine, St James's University Hospital, Beckett Street, Leeds LS9 7TF, UK; e Division of Molecular Structure, MRC-National Institute for Medical Research, Mill Hill, London NW7 1AA, UK

**Keywords:** Cancer, FGFR, Clinical inhibitors, Resistance mutations

## Abstract

Fibroblast growth factor receptors (FGFRs) are recognized therapeutic targets in cancer. We here describe insights underpinning the impact of mutations on FGFR1 and FGFR3 kinase activity and drug efficacy, using a combination of computational calculations and experimental approaches including cellular studies, X-ray crystallography and biophysical and biochemical measurements. Our findings reveal that some of the tested compounds, in particular TKI258, could provide therapeutic opportunity not only for patients with primary alterations in *FGFR* but also for acquired resistance due to the gatekeeper mutation. The accuracy of the computational methodologies applied here shows a potential for their wider application in studies of drug binding and in assessments of functional and mechanistic impacts of mutations, thus assisting efforts in precision medicine.

## Highlights

• Point mutations in FGFR can result in distinct signatures for drug efficacy.
• Gatekeeper mutations in FGFR1 and FGFR3 retain sensitivity to TKI258.
• Theoretical assessments of drug binding and allosteric effects show high level of accuracy.

## 1 Introduction

Fibroblast growth factors and their receptors (FGFs and FGFRs) play a critical role in many physiological processes including embryogenesis, wound healing, inflammation and angiogenesis as well as adult tissue homeostasis (Beenken and Mohammadi, 2009). Compelling evidence also implicates activation of FGFRs (FGFR1–4) in pathogenesis of several developmental syndromes and a broad range of human malignancies. FGF/FGFR signalling contributes to tumour generation and progression through activating *FGFR* genomic alterations (driver point-mutations, fusions and amplifications) (Greulich and Pollock, 2011, Wesche et al., 2011, Sabnis and Bivona, 2013), as a positive regulator of tumour neoangiogenesis (Turner and Grose, 2010) and as a mediator of resistance to endocrine (Turner et al., 2010) and targeted therapies to related oncogenic pathways, in particular to signalling by other receptor tyrosine kinases such as epidermal growth factor receptor (EGFR) (Wilson et al., 2012, Crystal et al., 2014).

The involvement of FGF/FGFRs in the pathology of many cancer types provides a strong rationale for development of effective agents for these targets and a large effort to develop FGFR inhibitors as anticancer treatments is underway (Brooks et al., 2012, Dieci et al., 2013). Some of the FGFR inhibitors such as TKI258 (dovitinib), levatinib, brivanib and AP24534 (ponatinib) also target a subset of other tyrosine kinases while AZD4547, PD173074, BGJ398 and JNJ-*493 appear to be selective for FGFR1–3. For the compounds already in clinical trials, important issues include optimising the management of emerging toxicity profiles and anticipated intrinsic target resistance as well as designing further trials to best match the target alterations with the proposed drug action. One of the bottlenecks in achieving such precision therapies is the lack of suitable approaches to functionally interpret vast quantities of genomic data. In particular, for FGFRs there are hundreds of mutations found in tumour samples (Greulich and Pollock, 2011, Wesche et al., 2011, Sabnis and Bivona, 2013) and their impact on FGFR activation cannot be predicted based on crystallographic insights alone; this is in part due to the considerable scope for allosteric effects inherent to protein kinases (Meharena et al., 2013).

Furthermore, the inhibitor binding can also be altered by various mutations. Prime examples are acquired intrinsic resistance mutations that have marred the success of tyrosine kinase inhibitors (TKIs) such as gefitinib, erlotinib and imanitib, prompting efforts for second- and third-line treatments (Daub et al., 2004, Azam and Daley, 2006, Gibbons et al., 2012). One resistance mechanism common to many kinase inhibitors is the mutation of the so-called “gatekeeper” residue that remains the most frequently detected drug-resistance mutation in the clinic. Examples include resistance to TKIs targeting breakpoint cluster region-abelson tyrosine kinase (BCR-ABL) fusion in chronic myelogenous leukaemia (Gorre et al., 2001, Shah et al., 2002), EGFR in nonsmall cell lung cancer (Kobayashi et al., 2005, Pao et al., 2005), platelet-derived growth factor receptor (PDGFR) in hypereosinophilic syndrome (Cools et al., 2003), KIT in gastrointestinal stromal tumours (Tamborini et al., 2006) and echinoderm microtubule-associated protein-like 4-anaplastic lymphoma kinase (EML4-ALK) fusion in lung cancer (Choi et al., 2010b). Modelling in cell culture has also been successfully used to discover clinically relevant acquired resistance and the application of this approach to FGFR driven-cancer cells identified a gatekeeper substitution (Chell et al., 2013). Gatekeeper substitutions in FGFR have been also identified in clinical samples, however as primary cancer mutations rather than secondary mutations (Taylor et al., 2009, Shukla et al., 2012, Ang et al., 2015). Taking into account the widespread occurrence of acquired gatekeeper resistance in many kinases and initial laboratory and clinical observations for FGFR, occurrence of this phenomenon in FGFR is widely anticipated.

Further factors that can influence drug binding include, pre-existing mutations in the targeted kinase or subtle differences between closely related family members. This has also been documented for the FGFR family members (Brooks et al., 2012, Dieci et al., 2013, Byron et al., 2013) emphasizing the need for further characterisation that would inform treatment.

Here we apply a combination of approaches to address current limitations in assessing whether a specific mutation in a kinase domain affects the activity or alters drug sensitivity. We report striking differences between FGFR sequence variants with respect to the effect on kinase activity and the efficacy of tyrosine kinase inhibitors. Furthermore, we found that the gatekeeper variant, that enhances kinase activity, is not refractory to some of the inhibitors currently in clinical trials; in particular, TKI258 retained its efficacy towards FGFR1 and FGFR3 gatekeeper substitutions in vitro and in cells. These findings, supported by measurements of kinase activity, determination of binding constants and X-ray crystallography, are in good agreement with the values obtained independently by molecular dynamics simulations that also provide an in-depth insight into the allosteric communication between the mutated site and important functional motifs. The computational methods used here to calculate the binding and surface free energies could therefore have wider application in predicting the functional impact of disease mutations, drug binding and underpinning molecular mechanisms.

## 2 Methods

### 2.1 Protein Expression and Purification

All kinase domain constructs were cloned into pOPINS (OPPF, Oxford, UK) or pJ821 (DNA2.0, USA) and expressed in C41 (DE3) cells harbouring lambda phosphatase and human CDC37. Kinases were purified by a combination of Ni^2 +^-chelating, ion exchange and gel filtration chromatography. Protein purity was assessed by SDS-PAGE and preparations at > 95% homogeneity were used for kinase assays and crystallography.

### 2.2 Kinase Assay

The ADP-Glo (Promega) methodology was used for all kinase assays following manufacturers instructions. Unless otherwise stated in the text, each kinase assay consisted of 20 μL, containing Kinase Buffer (40 mM Tris-HCl, 20 mM NaCl, 20 mM MgCl_2_, 2 mM TCEP, 2 mM MnCl_2_, Na_3_VO_4_, 0.1 mg/mL BSA, pH 8.0), 250 μM ATP, 0.4 mg/mL polyGlu_4_Tyr substrate, 0.5 μM kinase domain and inhibitors in selected experiments. In various experiments the concentrations of these components were varied and this is stated in the text or figure legends. In order to calculate valid inhibition constants (K_i_), a number of kinetic parameters needed to be ascertained, in particular, the K_m_ of the kinases for ATP. A thorough Michaelis–Menten kinetic analysis was performed ensuring that all relevant parameters were in the linear range and that the appropriate concentrations of ATP, substrate and kinase were adhered to. All data were processed using Graphpad Prism and parameters presented in Supplemental Tables. Classical enzyme competitive inhibition approaches were found to be unsuitable to generate inhibition constants and therefore the Morrison approach (the quadratic velocity equation for tight-binding substrates) was utilized (Morrison, 1969).

### 2.3 Crystallisation and Crystallography

Crystals of FGFR1-2c Apo, native FGFR1-2c with TKI258 (FGFR1-2c TKI258) and FGFR1-2c^V561M^ with TKI258 (FGFR1-2c^V561M^ TKI258) were grown by both hanging and sitting drop methods in condition 20% PEG 5 K MME, 0.1 M Tris, pH 7.5, 0.2 M ammonium sulphate. X-ray diffraction data were recorded at Diamond Light Source in stations I02, I03 and I24 for FGFR1-2c Apo, FGFR1-2c TKI258 and FGFR1-2c^V561M^ TKI258 respectively. The data were auto-processed using autoprocessing tools Xia2 (Winter et al., 2013) and FAST_DP (uses XDS (Kabsch, 2014)) at Diamond Light Source. The unmerged output from XDS was taken for each of the dataset and scaled using Aimless (Evans and Murshudov, 2013) software suite from CCP4 package (Winn et al., 2011). The FGFR1-2c Apo, FGFR1-2c TKI258 and FGFR1-2c^V561M^ TKI258 were scaled to a high resolution of 2.3 Å, 1.96 Å and 1.96 Å units respectively. Please refer to Supplemental Table S5, X-ray data processing statistics. Phasing, refinement and structure validation are described in the Supplemental Material.

### 2.4 Computational Calculations

The binding and the surface free energy changes were calculated using multiple thermodynamic integration simulations based on methodology described in (Wan and Coveney, 2011). Further improvements and enhanced-sampling molecular dynamic simulations (parallel-tempering metadynamics) were according to (Bussi et al., 2006); the algorithms were previously used in calculating the conformational free energy surfaces of EGFR (Sutto and Gervasio, 2013), c-SRC and ABL (Lovera et al., 2012). Computational calculations are described further in Supplemental Material.

### 2.5 PDB ID Codes

The refined and validated structures of FGFR1-2c Apo, FGFR1-2c TKI258 and FGFR1-2c^V561M^ TKI258 have been submitted to Protein Data Bank (PDB) and their PDB ID codes are 4UWY, 4UWZ and 4UX0: 4UWZ, 4UX0, respectively.

## 3 Results

### 3.1 Kinase Activity and Inhibition of FGFR Variants by Non-selective and Selective Inhibitors

**Fig. 1:**
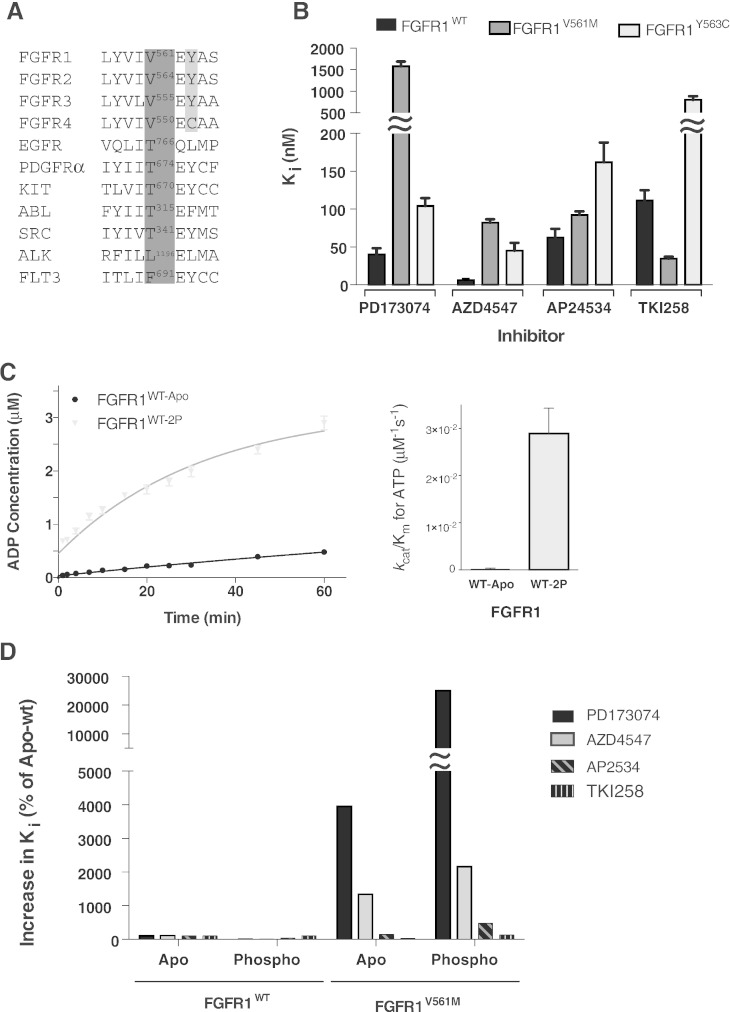
Kinase activity and inhibition of FGFR1 variants. A. Amino acid alignment of part of the hinge region of the FGFR family and a number of other tyrosine kinases with reported gatekeeper mutations. The gatekeeper residue in each case is numbered and bordered in dark grey. The FGFR 1 to 3 tyrosine that is a cysteine in FGFR4 is highlighted in light grey. B. Histogram showing the inhibition constants (K_i_) for four selected compounds upon FGFR1 kinase domain protein, wild type and the indicated mutants. The data were generated from enzyme kinetic analyses using various concentrations of inhibitors and fitting using the Morrison equation within Graphpad Prism. Each data point was repeated in duplicate and the standard error of the mean is presented on each bar. C. Left panel: comparison of the activity of FGFR1 apo (WT-apo) and phosphorylated (WT-2P) kinase domain protein produced using the ADP-Glo Assay. Each data point was produced in triplicate and the standard errors are indicated. Right panel: comparison of the enzyme efficiency (*k*_cat_/K_m_) of FGFR1 apo and phosphorylated protein. Parameters were generated through Michaelis–Menten kinetic experiments and analysed using Graphpad Prism software. D. Histogram showing the change in inhibition constants for phosphorylated FGFR^WT^ and apo and phosphorylated FGFR1^V561M^ when normalised to apo FGFR1^WT^ values (taken as 100%) for the indicated inhibitors. The data in B–D are representative for 3–4 independent experiments. See also Supplemental Tables S1 and S2.

Mutations in the ATP binding pocket of protein kinases that prevent or reduce binding of inhibitory compounds most frequently occur at a particular residue in the hinge region between the N and C lobes, positioned to control access to a hydrophobic pocket that helps anchor kinase inhibitors to the active site. This, so called, gatekeeper residue corresponds to threonine in a number of kinases (including EGFR, PDGFR, KIT, ABL and SRC) but can also be other amino acid residues such as leucine, phenylalanine or valine (Fig. 1A). Among all FGFR members, the gatekeeper valine is conserved, however, the tyrosine residue in its proximity in FGFR1-3 corresponds to a cysteine in FGFR4 (Fig. 1A). FGFR4 is known to have reduced sensitivity to several inhibitors and the position of the tyrosine to cysteine replacement (close to the gatekeeper) is likely to contribute to such a difference. For the analyses of inhibitor sensitivity, we focused on a structurally well-defined kinase domain (KD) of FGFR1 (Mohammadi et al., 1996, Bae et al., 2010, Norman et al., 2012), with the gatekeeper V561M variant and Y563C variant (corresponding to the replacement found in FGFR4) generated in the context of FGFR1.

TKI258 and AP24534 are among several pan-tyrosine kinase inhibitors that include FGFR in their panel of targets. They are currently being tested in preclinical and clinical studies for their anti-tumour activity through the direct inhibition of FGFR (Andre et al., 2013, Konecny et al., 2013, Motzer et al., 2014, Gozgit et al., 2012, Mazzola et al., 2014). A selective FGFR1-3 inhibitor PD173074 has been widely used in preclinical studies while AZD4547, that also selectively targets FGFR, is in several phase I and phase II clinical trials (Ye et al., 2014, Dutt et al., 2008, Lamont et al., 2011, Lei et al., 2014, Liu et al., 2014, Ramsey et al., 2013, Xie et al., 2013, Dieci et al., 2013). Analysis of these four inhibitors in an in vitro kinase assay showed that in addition to the expected differences in their potency towards the WT FGFR1, different inhibitors had altered efficacy towards V561M and Y563C variants (Fig. 1B, Supplemental Table S1). The Y563C replacement resulted in reduced sensitivity to all tested inhibitors with the most pronounced effect on inhibition by TKI258 (about 7-fold). Interestingly, the gatekeeper mutation V561M resulted in a clear reduction of sensitivity to PD173074 and AZD4547, a marginal effect on sensitivity to AP24534 and an increase in inhibition by TKI258 compared to wild type. Thus, these two closely positioned point mutations have specific signatures for inhibitor sensitivity. The data also show, that the gatekeeper mutation, that would potentially result from treatment with compounds such as PD173074 or AZD4547, has a similar or better response compared to the WT to some other inhibitors that target FGFR, namely, AP24534 and TKI258.

Further study of the V561M gatekeeper mutation examined how the phosphorylation state of the FGFR1 KD affects sensitivity to the selected inhibitors. Previous studies had identified phosphorylation of two tyrosine residues (Y653 and Y654) as the key step in the shift towards an activated form of FGFR1 (Furdui et al., 2006). We generated this phospho-form (WT-2P) of FGFR1 and confirmed an increase in kinase activity (Fig. 1C, Supplemental Table S2). Comparison of non-phospho and phosphorylated forms of the WT and gatekeeper has shown that the relative change in potency of the inhibitors was similar to that observed for the non-phospho forms (Fig. 1D, Supplemental Table S1). The data also show that the phospho form of the V561M variant was less sensitive to all inhibitors with the sensitivity to TKI258 being similar to that observed for the phospho WT (K_i_ values of 142.0 ± 15.7 and 121.2 ± 5.4, respectively; Supplemental Table 1).

We also compared actual K_i_ values for each inhibitory compound (Fig. 1B, Supplemental Table S1). This shows that AZD4547, due to its potency, could theoretically, be effective towards the gatekeeper substitution even when its K_i_ is increased (from 6.1 nM to 82.1 nM). However, further considerations involving parameters relevant for drug administration (such as effective dose and side effects) or other consequences that mutations could have on the global state of the harbouring cell, need to be taken into account.

### 3.2 Effect of the Gatekeeper Mutation on Direct Binding of FGFR Inhibitors

The potency among the tested inhibitors on the gatekeeper variant showed the biggest difference between PD173074 and TKI258, where the change of up to 225-fold reduction contrasts to no change or even higher efficacy towards the non-phospho form (Supplemental Table S1). To understand different binding modes that could underlie the observed difference in inhibition, we performed theoretical calculations of binding free energies in parallel with direct experimental measurements using ITC and structural analysis by X-ray crystallography.

**Fig. 2:**
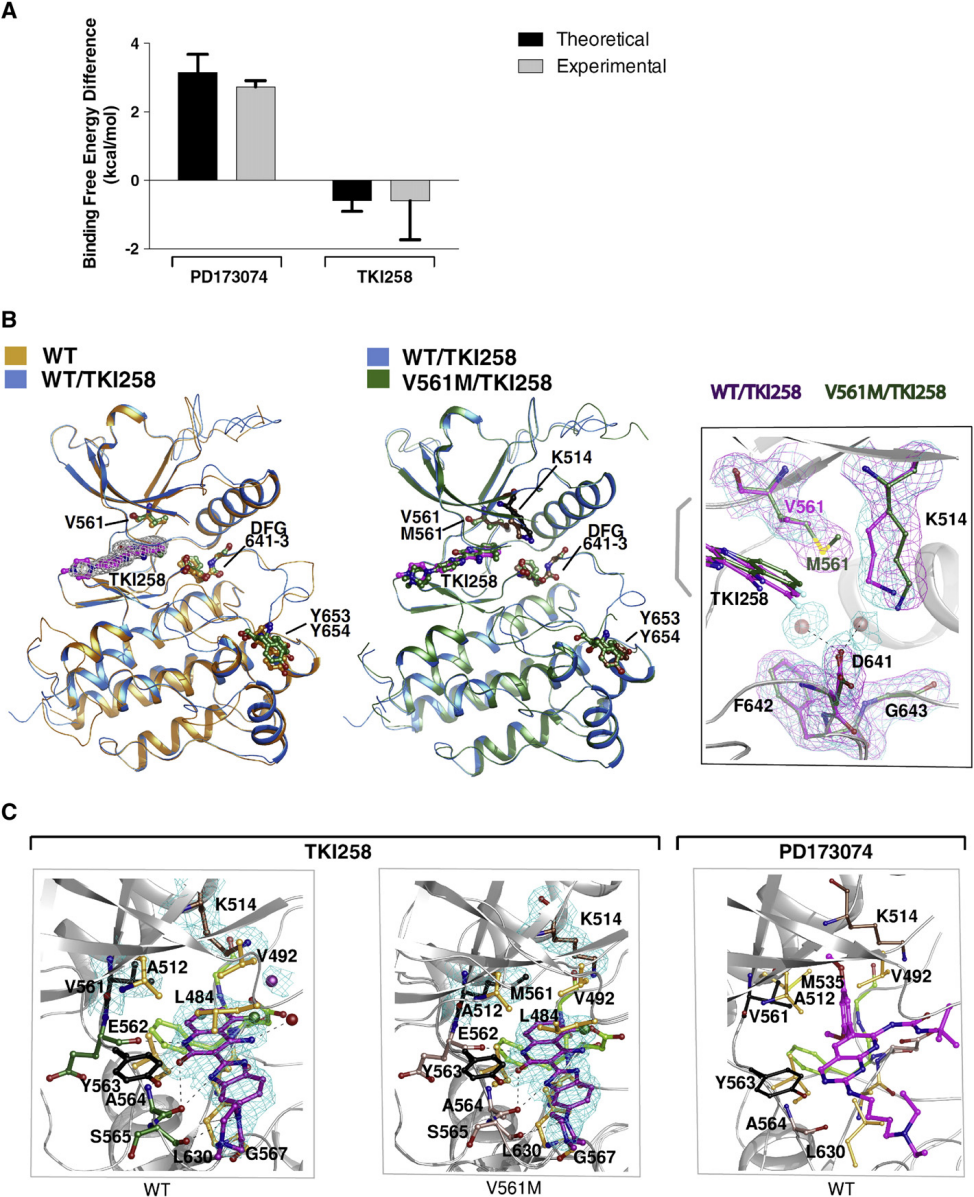
Binding of FGFR-inhibitors to wild type (WT) and V561M FGFR1 KD. A. Histogram comparing the theoretical (generated through molecular dynamic simulations) and the experimental (generated through ITC) difference in free energy of the indicated inhibitors binding to FGFR1^WT^ and FGFR^V561M^. See also Supplemental Table 3. B. Superposition of apo WT (orange) and WT FGFR1 KD bound to TKI258 (marine) (left panel) and superposition of TKI258-bound WT (marine) and TKI258-bound V561M FGFR1 KD (forest green) (middle panel) are shown as ribbon representations. Electron density map (2Fo-Fc) contoured at 1.0 σ for TKI258 is shown for the WT (left panel). TKI258 bound to WT FGFR1 KD and V561M FGFR1 KD are coloured in magenta and forest green respectively. Several key residues, including DFG motif, gatekeeper residue and Y 653,654 are indicated. The right panel features superposition of TKI258 bound to WT FGFR1 KD and FGFR1 KD V561M showing the gatekeeper residue V561 and M561, and K514 displacement in FGFR1 KD V561M structure. Electron density map (2Fo-Fc) contoured at 1.0 σ is shown for the gatekeepr, DFG motif and Lys514 residues. The solvent molecules in WT FGFR1 KD at the active site are shown as red spheres along with electron density map (2Fo-Fc) controured at 1.0 σ. C. Ball-and-stick representation of TKI258 bound WT FGFR1 KD and FGFR1 KD V561M is shown in the left and middle panel, respectively. Key residues are shown and labelled. Electron density map (2Fo-Fc) contoured at 1.0 σ is shown for TKI258, gatekeeper residue V/M561 and K514. Solvent molecule interacting with TKI258 is shown as red sphere. Hydrogen bonds are shown as black dashed lines. Fluoride atom is coloured in green. Ball-and-stick representation of WT FGFR1 KD bound PD173074 (PDB: 2FGI) and its interacting residues are shown in the right panel. In all panels DFG motif is coloured in lime, Y563, V/M561 in black, residues involved in hydrogen bonding interactions are coloured in salmon or green and residues involved in hydrophobic interaction and van der Waals contacts are in orange.

The binding free energy change upon gatekeeper mutation was estimated using multiple thermodynamic integration (TI) simulations in both directions, i.e. alchemically mutating valine to methionine and vice versa (Table 1). The experimental results of ITC measurements, including Kd, ∆H, T∆S and ∆G, are shown in Supplemental Table S3. For PD173074, the calculated binding free energy difference ∆∆G (3.25 ± 0.42 kcal/mol) matches the experimental value (2.73 ± 0.18 kcal/mol) from ITC experiments. Both calculation and experiment demonstrate that the gatekeeper mutation V561M in FGFR1 reduces the binding affinity for PD173074. The same approach was applied to the binding of TKI258 and here again the calculated binding free energy difference and the experimental values were comparable (theoretical ∆∆G of − 0.55 ± 0.12 kcal/mol compared to experimental − 0.60 ± 1.14 kcal/mol), showing somewhat better binding to the gatekeeper. The accuracy of our TI simulations is further supported by another example (free binding energy differences of PD173074 for the Y563C variant) with good agreement with experimental values (Supplemental Tables S3 and S4). The comparison of experimental values and computational calculations is summarised in Fig. 2A.

**Table 1:** Binding free energy difference ΔΔ*G*^*binding*^ = Δ*G*_*mut*_^*binding*^ − Δ*G*_*WT*_^*binding*^ (kcal/mol).

Simulation	Energy	Rep1	Rep2	Rep3	Rep4	Rep5	Average	ΔΔ*G*_*theor*_	ΔΔ*G*_*theor*_^*avg*^	ΔΔ*G*_*exp*_
*FGFR1^V561M^ with PD173074*										
Forward	Δ*G*_*protein*_^*alch*^	1.03	0.46	1.08	0.60	0.96	0.83	3.66	3.25 (0.42)	2.73 (0.18)
	Δ*G*_*complex*_^*alch*^	− 2.90	− 2.28	− 3.28	− 3.07	− 2.63	− 2.83			
Reverse	Δ*G*_*protein*_^*alch*^	0.73	0.51	0.77	1.31	0.97	0.86	2.83		
	Δ*G*_*complex*_^*alch*^	− 2.35	− 1.54	− 1.95	− 1.70	− 2.31	− 1.97			

*FGFR1^V561M^ with TKI258*										
Forward	Δ*G*_*protein*_^*alch*^	1.03	0.46	1.08	0.60	0.96	0.83	− 0.43	− 0.55 (0.12)	− 0.60 (1.14)
	Δ*G*_*complex*_^*alch*^	0.68	1.18	1.53	1.69	1.20	1.26			
Reverse	Δ*G*_*protein*_^*alch*^	0.73	0.51	0.77	1.31	0.97	0.86	− 0.66		
	Δ*G*_*complex*_^*alch*^	1.48	1.39	1.84	0.66	2.22	1.52			

Values in parentheses are uncertainties of the binding free energy changes. For calculation, they are simply half the differences of the forward and reverse results, while for the experiment, they are standard deviations of repeated measurements.

Of the two inhibitors analysed in our theoretical study, only the structure of PD173074 bound to the WT FGFR1 KD has been available (Mohammadi et al., 1998). To extend the structural analysis, we solved the structures of the WT FGFR1 KD without and with bound TKI258 and of FGFR1 KD V561M variant with TKI258 at 2.3, 1.96 and 1.96 Å resolution respectively (Supplemental Table S5). TKI258 binds in the hydrophobic pocket at the ATP-binding cleft and is stabilised by hydrogen bonds, hydrophobic and van der Waals contacts (Fig. 2B and C, Supplemental Table S6A). The drug engages the enzyme with a total contact area of ~ 60 Å^2^. Five hydrogen bonds (involving residues E562, A564 and S565) and 10 amino acid residues involved in hydrophobic interactions provide a direct contact between WT FGFR1 and TKI258 and stabilises the drug–protein complex (Fig. 2C and Supplemental Fig. S1A). One water molecule is found to interact with atom N29 of TKI258. The structures of TKI258 bound WT FGFR1 KD and FGFR1 KD V561M superposes on apo WT FGFR1 KD with an overall root mean square deviation (rmsd) value of 1.02 (287 amino acid residues) and 1.01 (286 amino acid residues) Å units, while the drug bound WT FGFR1 KD and FGFR1 KD V561M superpose on each other with an rmsd value of 0.36 Å units (293 amino acid residues) (Supplemental Table S6C). The low rmsd values and illustrated superimposed structures in Fig. 2B (left and middle panel) suggest that the binding of TKI258 to FGFR1 KD does not alter the enzyme structure.

TKI258 binds more tightly to the FGFR1 KD V561M (K_i_ ~ 35 nM) while binding of PD173074 (K_i_ ~ 1580 nM) is greatly obstructed (Supplemental Table S1); structural insights highlight the underpinning differences (Fig. 2C). TKI258 binding is strengthened by the gatekeeper residue, M561, at its binding site by increasing the number of hydrophobic contacts. The total number of van der Waals contacts for WT FGFR1 KD and FGFR1 KD V561M are 28 and 37, respectively. In addition, M561 pushes two water molecules and K514 out of the binding cleft, which was observed in the TKI258-WT FGFR1 KD structure (Fig. 2B, right panel and C, left and middle panels). Thus in the gatekeeper mutant, the polar charge contribution of K514 and two water molecules are void at the binding cleft leading to a stronger affinity of TKI258 to the kinase. The total number and strength of van der Waals contacts for TKI258 in FGFR1 KD V561M increases (with S^δ^ and C^ε^ atoms from M561) compared to V561 in WT FGFR1 KD. In contrast to TKI258, the structure of PD173074 bound to the WT FGFR1 KD (PDB: 2FGI) shows that the drug binds deep inside the ATP binding cleft and forms a very strong hydrophobic interaction with M535 and V561 (Fig. 2C). M535 forms a lock-and-key type of interaction with the dimethoxy group of the compound while the phenyl ring is well stabilised by a stacking interaction from the gatekeeper residue V561. Consequently, PD173074 exhibits very weak affinity (Supplemental Table S1) to the FGFR1^V561M^ gatekeeper mutant due to its close proximity to the gatekeeper residue M561. The S^δ^ and C^ε^ atoms of M561 in FGFR1^V561M^ would cause steric clashes with the phenyl ring and push the ligand away and out from the binding pocket as the major contributing factor to greatly reduced sensitivity to this inhibitor.

Further structural considerations based on differences between inactive and active forms of FGFR1 and other FGFR KD structures (PDB: 3GQI, 2PY3, 2PVF, 2PSQ, 4K33) support the observed lower efficacy of TKI258 towards the phosphorylated, active form (Supplemental Figs. S2 and S3). Upon stimulation FGFR KD undergoes major sequential structural changes. These changes involve the movement of the N-lobe towards the C-lobe of KD,, aligning of residues within the catalytic and regulatory (hydrophobic) spines impact on “molecular brake” and the opening of A- loop to accommodate ATP (PDB: 3GQI). As a consequence the backbone and side chain atoms of FGFR residues that interact with TKI258 show a significant rearrangement. The result is a reduction in accessible surface area for the ligand, leading to fewer/weaker hydrogen bonding interactions and van der Waals contacts (Supplemental Fig. S3).

### 3.3 Effect of the Gatekeeper Mutation on FGFR1 Kinase Activity

**Fig. 3:**
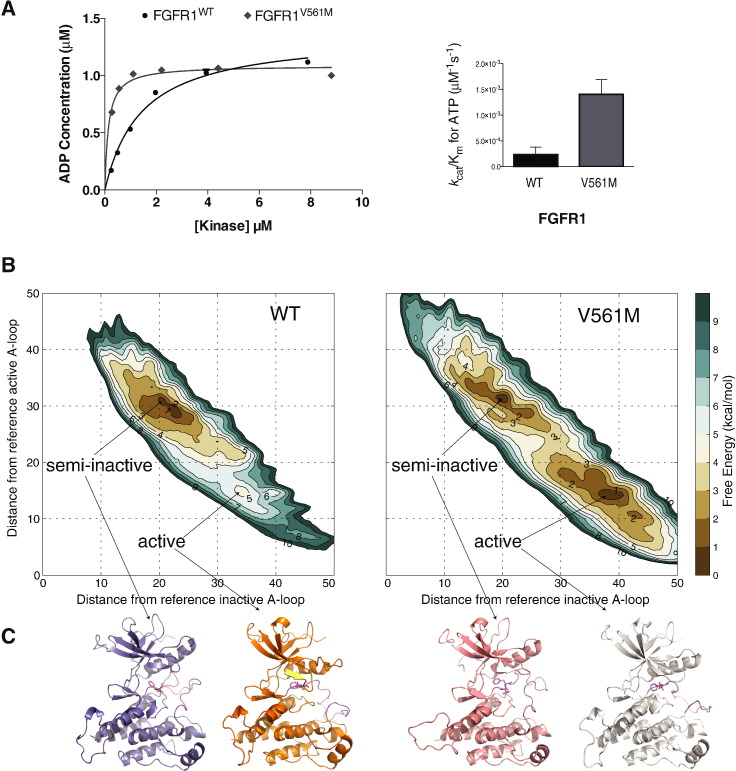
Impact of the gatekeeper substitution V561M on the kinase activity and overall structural changes of FGFR1 KD. A. Left panel: comparison of the activity of FGFR1^WT^ and FGFR^V561M^ produced using the ADP-Glo Assay. Increasing concentrations of kinase were incubated for 60 min and the level of ADP produced analysed. Data were fit with a hyperbolic model (for presentation purposes only) using Graphpad prism. Each data point was produced in triplicate and the standard errors are indicated. Right panel: comparison of the enzyme efficiency (*k*_cat_/K_m_) of FGFR1^WT^ and FGFR^V561M^. Parameters were generated through Michaelis–Menten kinetic experiments and analysed using Graphpad Prism software. See also Supplemental Tables S1 and S2. B. The free energy surfaces of the activation of WT-FGFR and V561M-FGFR are shown as a function of the distance from the reference inactive A-loop conformation (CV1) and to the distance from the reference inactive A-loop conformation (CV2). The contour lines are drawn every 1 kcal/mol. C. Ribbon representations of the proposed structures of FGFR1 WT and V561M coming from the full atomistic modelling molecular dynamics approach in the semi-inactive and active forms.

Analyses of gatekeeper threonine to bulkier side chain (methionine or isoleucine) substitutions in other kinases, in particular in SRC and EGFR, have shown that the mutations result in an enhancement of the kinase activity (Azam et al., 2008). In FGFR kinases the corresponding wild type amino acid residue is valine and replacement with a larger hydrophobic residue could have a similar effect. Direct comparison of the WT and V561M FGFR1 variants in an in vitro kinase assay shows higher activity of the gatekeeper and a 6-fold increase of the *k*_cat_/K_m_ ratio for ATP (Fig. 3A). A spatially close Y563C substitution resulted in the reduction of kinase activity (Supplemental Table S2).

The same conclusion, namely an increase in kinase activity of the gatekeeper V561M variant, was independently reached using enhanced-sampling parallel tempering metadynamics molecular dynamics (MD) simulations (Fig. 3B and C). Similar data were obtained using TI simulations (Supplemental Table S7). Using these two well-established methods for calculating free energies (TI simulations and parallel tempering metadynamics), together with two of the best protein force fields (Amber ff99SBildn and CHARMM22*), we find theoretical agreement with respect to the activating nature of the mutation and the associated mechanism of action. This information could not be obtained based only on static crystal structures of the WT and gatekeeper variants with TKI258.

The reconstruction of the fully converged free energy surface (FES) of the activation transition shows a clear stabilizing effect of the mutation on the active state of FGFR as compared to WT FGFR (Fig. 3B and C). Indeed, the active state of the non-phosphorylated WT FGFR is a high-energy minimum (ΔG ~ 5 kcal/mol). The most stable conformation corresponds to a partially deformed activation loop, resulting in a conformation intermediate between the active and the fully inactive one that we refer to as semi-inactive; a similar semi-inactive state has recently been reported in EGFR (Shan et al., 2012, Sutto and Gervasio, 2013). In contrast, the V561M mutation both stabilises the activation loop in the open, fully active conformation by 4 kcal/mol with respect to the WT FGFR, and lowers the free energy barrier between the two states. The populations of the semi-inactive and active activation loop conformations are similar in the case of the mutant, separated by a relatively low (~ 3 kcal/mol) free energy barrier.

MD simulations also suggest the mechanism by which the methionine allosterically induces a large shift in the energy landscape of the FGFR1 KD. Consistent with the insights from the crystal structure of FGFR1 V561M variant (Fig. 2B), MD simulations also depict the change where the methionine fills up the hydrophobic pocket adjacent to the active site stabilizing it (Supplemental Fig. S4). In the active basin of the FGFR1^V561M^, the interactions of M561 involve the hydrophobic residues of the αC helix M535 and L547, the highly conserved residues surrounding the ATP binding pocket V492, I545 and L630, and the L547 and F642 of the hydrophobic spine. The αC helix and the E531:K514 salt bridge are stably formed in both the active and inactive basins of both FGFR1 and FGFR1^V561M^ kinases. Together, these interactions result in stabilization of the A-loop in the active conformation and impact on auto-inhibition due to “molecular brake”.

### 3.4 Gatekeeper Mutation and Oncogenic Variants of FGFR3

FGFR1, FGFR2 and FGFR3 all contribute to the generation of tumours in diverse cancer types that are in clinical trials for both selective and non-selective FGFR-inhibitors (Greulich and Pollock, 2011, Wesche et al., 2011, Sabnis and Bivona, 2013, Dieci et al., 2013). The V561M substitution in FGFR1 analysed here (Fig. 1, Fig. 2, Fig. 3) is unlikely to occur in FGFR2 as it would require two point mutations; instead, single point mutations in the codon corresponding to the gatekeeper residue would result in V564F, V564I or V564L mutations. Taking into account that subtle differences in the ATP binding pocket (as well as overall differences of the structural context) could have a big impact on the inhibitor binding, it could be expected that outcomes of such substitutions in FGFR2 could be different as supported by recent observations for FGFR2 V564I (Byron et al., 2013). In contrast, the FGFR1-equivalent substitution in FGFR3 (V555M) is highly likely to occur and was previously identified in the context of a cancer cell line (multiple myeloma KMS-11) dependent on an FGFR3 Y373C driver mutation (Chell et al., 2013). However, the effects of the gatekeeper mutation on its own, or in combination with other FGFR3 driver mutations, have not been analysed. Furthermore, in addition to in vitro data, the effectiveness of different FGFR inhibitors on cells transformed by oncogenic variants of FGFR with the additional gatekeeper mutation needs to be assessed.

**Fig. 4:**
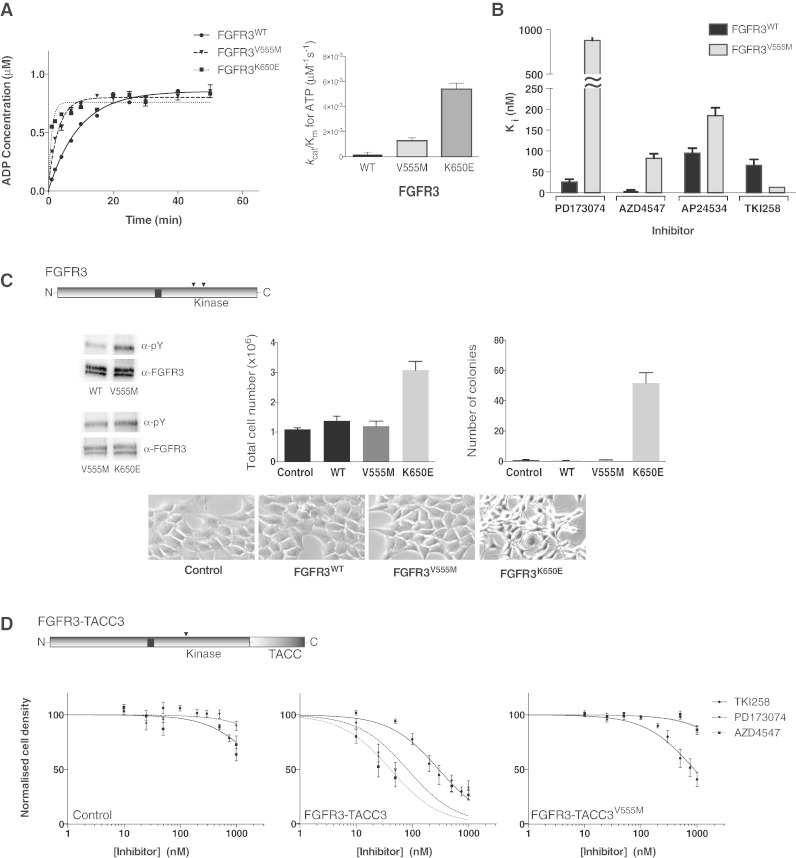
Kinase activity and inhibition of FGFR3 variants assessed in vitro and in cells. A. Left panel: Comparison of the activity of FGFR3 wild type, V555M and K650E kinase domain protein produced using the ADP-Glo Assay. Data were fit with a one-phase association model (for presentation purposes only) using Graphpad prism. Each data point was produced in triplicate and the standard errors are indicated. Right panel: Comparison of the enzyme efficiency (*k*_cat_/K_m_) of FGFR3 wild type, V555M and K650E kinase domain protein. Parameters were generated through Michaelis–Menten kinetic experiments and analysed using Graphpad Prism software. See also Supplemental Table S2. B. Histogram showing the inhibition constants (K_i_) for four selected inhibitors upon FGFR3 kinase domain, wild type and V555M mutant. The data were generated from enzyme kinetic analyses using various concentrations of inhibitors and fitting using the Morrison equation within Graphpad Prism. Each data point was repeated in duplicate and the standard error of the mean is presented on each bar. See also Supplemental Table S1. C. Diagram at the top shows intact FGFR3 with the positions of V555 and K650 indicated in the kinase domain; in FGFR3 IIIb these residues have been assigned as V557 and K652, respectively. Top panel, left shows a representative Western blot analysis (using indicated antibodies) of samples from NIH3T3 cells stably expressing wild type (WT), V555M or K650E variants after immuno-precipitation with anti-FGFR3 antibody. Representative experiment showing the total cell numbers reached on day 7 by cells expressing wild type (WT) or mutant (V555M or K650E) FGFR3, compared to control cells (vector alone) is presented in the middle panel. Fold difference in the number of colonies formed in soft agar by cells expressing wild type or mutant FGFR3 and control cells is shown in the right panel. Bottom panels show morphology of NIH3T3 cells expressing the indicated constructs. D. Diagram at the top shows FGFR3-TACC3 fusion protein with the position of V555 in the kinase domain and the TACC3 portion at the C-terminus indicated. Control (vector only), FGFR3-TACC3 and FGFR3-TACC3^V555M^ stable cell lines were treated with increasing concentrations of the indicated inhibitors. Cell viability was assessed at 72 h. Data were fitted with a log(inhibitor) vs. normalised response equation within Graphpad Prism. The data in A and B are representative for 4 independent experiments and in C and D for 2 independent experiments.

Analysis of the kinase activity of the FGFR3 V555M gatekeeper variant shows that, as in FGFR1, the gatekeeper mutation resulted in enhanced activity. Compared to the most potent activating point mutation in FGFR3, K650E, this increase is less pronounced (*k*_cat_/K_m_ comparison) (Fig. 4A, Supplemental Table S2). Determination of changes in K_i_ values for the four selected inhibitors (Fig. 4B) is also in good agreement with the data obtained for FGFR1 V561M (Fig. 1B, Supplemental Table S1). Furthermore, the direct assessment of TKI258 binding to FGFR3 using NMR is consistent with the structure of FGFR1 KD/TKI258 complex solved by X-ray crystallography (Supplemental Fig. S1B). Based on this close similarity, it is expected that the phospho-form (or activated forms) of FGFR3 would also be somewhat less sensitive to TKI258.

The comparison of stable NIH 3T3 cell lines expressing FGFR3 WT, FGFR3 V555M or FGFR3 K650E variants has shown that the phosphorylation of FGFR3 V555M is increased compared to the WT but is not as high as observed for the activated form FGFR3 K650E (Fig. 4C). Further analysis of these cell lines revealed that, unlike the oncogenic FGFR3 K650E variant, the FGFR3 V555M gatekeeper on its own lacks potential to increase growth rate or induce anchorage independent growth and a transformed phenotype in NIH 3T3 cells (Fig. 4C).

Another recently discovered, clinically important FGFR3 oncogenic drivers are the FGFR3-TACC3 fusion proteins (Williams et al., 2013, Singh et al., 2012); one such fusion protein is depicted in Fig. 4D. This FGFR3-TACC3 protein includes a large portion of the WT FGFR3 IIIb sequence covering the entire KD and lacking only a small portion at the C-terminus that is replaced by TACC3. Importantly, cancer cell lines (RT112 and RT4, bladder) and transformed cell lines harbouring such fusion proteins have been extensively studied for inhibitor sensitivity both in culture and in animal models (Lamont et al., 2011, Singh et al., 2012). NIH 3T3 cells expressing FGFR3-TACC3 fusion protein are selectively inhibited by FGFR inhibitors such as PD173074 compared to cells expressing FGFR3 WT that are unaffected (Supplemental Fig. S5A) in agreement with observations using cancer cell lines (Lamont et al., 2011). Introduction of the V555M gatekeeper to the FGFR-TACC3 construct retained the general oncogenic properties including the transformed phenotype and higher growth rate of the NIH 3T3 cell line (Supplemental Fig. S5B).

Treatment of control NIH 3T3 cells with PD173074, AZD4547 or TKI258 had very little or no effect within the concentration range up to 1 μM (Fig. 4D, left panel). AP24534 was not included in this study due to inhibitory effects (over 60% at 1 μM concentration) of this multi-kinase drug under control condition in this particular cell line. AP24534 inhibits some non-receptor (ABL, LYN and SYK; IC50 about 0.2–0.4 nM) and receptor tyrosine kinases FGFR, vascular endothelial growth factor receptor (VEGFR) and PDGFα (IC50 values 1–2.5 nM). In contrast, TKI258 targets several related tyrosine kinase receptors [FGFR, FMS-like tyrosine kinase-3 (FLT3), KIT and VGFR with IC50 values within a range of 1–10 nM] (Dieci et al., 2013). The effect of PD173074, AZD4547 or TKI258 on the FGFR3-TACC3 fusion NIH3T3 cell line reflected the K_i_ values determined using the kinase assay in vitro, although within a narrower range. AZD4547 was most effective, followed by PD173074 and less inhibitory TKI258 (Fig. 4D, middle panel). The NIH 3T3 cell line expressing the FGFR3-TACC3 V555M variant was affected only by TKI258 (Fig. 4D, right panel). Surprisingly, AZD4547 was completely ineffective in this cell line, contrasting the binding constants observed in vitro where the gatekeeper mutation V555M was analysed as the only alteration within the FGFR3 KD (K_i_ of about 80 nM). Although further analyses of more FGFR3 variants in a cellular context would be needed to address such complexity, this observation is consistent with the previous data showing that KMS-11 cells harbouring FGFR3 Y373C, V555M variant were only marginally affected by 1 μM AZD4547 and were maintained in the presence of a closely related AZ8010 compound at the concentration of 3 μM (Chell et al., 2013). Overall, our findings, shown in Fig. 4D, highlight the potency of TKI258 to inhibit oncogenic variants of FGFR3 incorporating the V555M mutation.

## 4 Discussion

The combination of experimental and theoretical approaches applied here provides new insights into the consequences of specific substitutions in FGFR1 and FGFR3 for the drug binding and kinase activity. In particular, our data provide structural and mechanistic insights into changes that accompany gatekeeper substitutions leading to kinase activation and their ability to be targeted by some but not all FGFR inhibitors. Importantly, the data highlight the potential of TKI258 and TKI258-like compounds as a second line treatment to anticipated resistance to several compounds currently in clinical use to target FGFR in cancer.

The activating effect of gatekeeper substitutions on kinase activity has been documented for several kinases including SRC, ABL and EGFR using structural and computational methodologies. There are only a few crystal structures available where the most common threonine gatekeeper residue is substituted by methionine (SRC T338M, EGFR T790M and ABL T315I) or isoleucine (SRC T341I) (Getlik et al., 2009, Zhou et al., 2009, O'Hare et al., 2009, Azam et al., 2008). MD simulations have been performed for EGFR T790M and together with experimental evidence show the activating effect on the EGFR kinase (Sutto and Gervasio, 2013). Furthermore, these previous findings and our data for FGFR1 V651M variant, including measurements of kinases activity, crystallography and MD stimulations (Fig. 1, Fig. 2, Fig. 3) reinforce a similar underlying mechanism. As first suggested for SRC T341I (Azam et al., 2008), the valine to methionine substitution in FGFR1 leads to exclusion of water molecules and a re-arrangement of K514 side chain potentiating the hydrophobic spine (that now includes M651) and facilitates coordination of ATP by K514 (Fig. 2B and C). MD simulations suggest a set of further interactions in the active basin of FGFR1^V561M^ and outline allosteric changes that result in a shift towards the active form, including an impact on “molecular brake”, a feature not present in most other kinases (Supplemental Fig. S4). Furthermore, resistance mutations can have an effect on drug binding not only by direct steric hindrance but also by allosterically modulating kinase dynamics as shown for EGFR T790M (Yun et al., 2008, Wan and Coveney, 2011, Sutto and Gervasio, 2013); this may also be a contributory factor in FGFR resistance.

We have shown that the gatekeeper valine to methionine substitutions in FGFR1 and FGFR3 resulted in reduction of sensitivity to selective inhibitors PD17307 and AZD4547; two other tested inhibitors, AP24534 and TKI258, retained their efficacy (Fig. 1, Fig. 2, Fig. 4, Table 1). While the multi-kinase, type II inhibitor AP24534 (ponatinib) has been specifically developed to target the gatekeeper mutation in the BCL-ABL fusion protein occurring in response to imatinib treatment of chronic myeloid leukaemia (O'Hare et al., 2009, Choi et al., 2010a), TKI258 (dovitinib) was originally developed as a novel class of RTK inhibitors targeting FLT3, KIT, VGFR and some PDGFR members, in addition to FGFR1-3 (Renhowe et al., 2009). TKI258 has been and continues to be tested in clinical trials including malignancies linked to *FGFR* genetic alterations (breast cancer, renal cell carcinoma and bladder cancer) and its other targets (such as KIT in gastrointestinal stromal tumours/GIST) (Andre et al., 2013, Konecny et al., 2013, Mazzola et al., 2014, Kang et al., 2013). Our findings that TKI258 can inhibit FGFR kinases incorporating gatekeeper substitutions (Fig. 1, Fig. 2, Fig. 4) suggest that it could be also considered as a second-line treatment following failure of imatinib in GIST patients because one of the frequent causes of such failure is the intrinsic resistance mutation in the related KIT target, affecting the gatekeeper residue (Tamborini et al., 2006).

Our data, including previously unavailable high-resolution crystal structure of TKI258 bound to a protein kinase, show that TKI258 is a type I inhibitor and binds to native FGFR1 without causing any structural alterations (Fig. 2B and C). Similar considerations apply to binding to FGFR3. When compared to type I FGFR1–3 selective inhibitors and type II inhibitor AP24534, TKI258 occupies a smaller region confined within the adenosine-binding pocket (Supplemental Fig. S6). Selective inhibitors PD173074, AZD4547 and NVP-BGJ398 (the latter two under clinical trials) share the 3,5-dimetoxy-phenil moiety and as shown for PD173074 and NVP-BGJ398 have closely overlapping binding pockets (Supplemental Fig. S6). Interestingly, FGFR1–4 are among only 15 out of 490 human protein kinases that have valine as the gatekeeper and the selective inhibitors are likely to involve an interaction with this uncommon residue (for example, V561 in FGFR1 interacts with PD173074 and NVP-BGJ398, Supplemental Fig. S6). Thus, it is possible that a shared single point mutation such as gatekeeper replacement would confer some degree of resistance to all these inhibitors. Despite a weaker binding to a phosphorylated, active form, more pronounced for the gatekeeper variant, TKI258 retains its inhibitory activity towards the gatekeeper replacement in FGFR1 and FGFR3 in vitro and in cells (Fig. 1, Fig. 2, Fig. 4). Furthermore, the availability of the high-resolution crystal structure with TKI258 (Fig. 2B and C) can guide further modifications to increase efficacy and selectivity for FGFR while retaining activity towards the gatekeeper mutation. Therefore, TKI258 or its derivatives may provide therapeutic opportunity not only for patients with genetic alterations in *FGFR* but also for those with acquired resistance to several selective FGFR-inhibitors.

Recently, a modification of the FGFR-specific inhibitor PD173074, has been reported that covalently targets a cysteine residue in the P-loop (Tan et al., 2014, Huang et al., 2015). The resulting compounds, in particular FIIN2 and FIIN3, proved effective towards mutations of the gatekeeper residue in all FGFR receptors (Tan et al., 2014). Compared to TKI258 the FIIN compounds occupy a space in the binding pocket that is closer to the side-chain residues of the gatekeeper replacements; however, due to its irreversible binding capability and additional stabilization FIIN retains efficacy towards these gatekeeper variants (Supplemental Fig. S7). Further development of FIIN compounds provides an alternative route towards effective second-line treatments.

Comparison of our data obtained using computational methods and findings obtained using experimental approaches, based on structural and biochemical characterisation, shows remarkable consistency (Figs. 2A and 3). In particular, the computational study (Table 1 and Supplemental Tables S4 and S7) emphasizes the importance of using ensemble MD simulations and thermodynamic integration calculations to overcome the insufficiencies of conformational sampling in single simulations, so as to generate accurate and reproducible results even in cases (such as binding of TKI258) where the free energy difference is small. Together with the enhanced sampling (Fig. 3), this methodology shows potential for wider application in studies of drug binding and in assessments of functional and mechanistic impacts of disease mutations. In turn, these approaches can improve the ability to perform molecular-based patient selection that would assist clinical trials and subsequent treatment.

In conclusion, compelling evidence supports the role of FGFRs as key drivers in the pathogenesis of diverse tumour types. Consequently, and coupled with the absence of any licensed therapeutics, there are intensive ongoing efforts to develop treatments targeting these receptors. These efforts also recognize the need to address the widespread problem of emerging resistance to current drugs, including frequent gatekeeper mutations in receptor tyrosine kinases. We identified that one of the drugs currently in clinical trials — TKI258, or its improved derivatives, could be used to target this type of resistance in FGFR and possibly in related targets. We also show that the computational calculations used here have wider application in assessing the best line of treatment based on genetic data.

## Authors' Contributions

M.K., T.D.B., and P.V.C. planned the project. T.D.B. and N.T. designed and performed experiments covering protein purification (T.D.B.), biophysical characterisation (T.D.B.), kinase assay (T.D.B.) and crystallography (N.T.) and analysed the data. S.W. and P.V.C. designed and S. W. performed computational calculations using multiple thermodynamic integration simulations. L. C. and F.L.C. designed and L. C. performed computational calculations using enhanced-sampling molecular dynamic simulations (parallel-tempering metadynamics) and analysed the data. H.K. performed NMR and analysed the data. M.A.K. and S.V.W. designed and S.V.W. performed generation and analysis of stable cell lines. P. A. performed molecular docking. N.T., T.D.B., M.K., S.W., L. C. and F.L.C. prepared figures and tables. M.K. wrote the manuscript and all authors read, corrected, and approved the final manuscript.

## Role of the Funding Sources

None of the funding sources had a role in the writing of the manuscript or the decision to submit it for publication.
