# Supplementary material for: The Effect of Mutations on Drug Sensitivity and Kinase Activity of Fibroblast Growth Factor Receptors: A Combined Experimental and Theoretical Study
Source: eBioMedicine. 2015 Feb 15;2(3):194–204. doi: 10.1016/j.ebiom.2015.02.009 (PMC4471147; doi:10.1016/j.ebiom.2015.02.009)
Supplement: Supplementary file 1 — Supplementary material. [file mmc1.pdf]

SUPPLEMENTAL MATERIAL

Supplemental Materials and Methods

Chemicals

All inhibitor compounds were purchased from Selleck Chemicals (Houston, USA). All other chemicals were purchased from Sigma (Poole, UK) unless otherwise stated in the text.

Molecular Biology, Protein expression and Purification

Mutations within all constructs were introduced using the site directed mutagenesis method (Agilent Technologies).

FGFR kinase domains were cloned into either pOPINS (OPPF, Oxford, UK) or pJ821 (DNA2.0, Menlo Park, USA) using In-Fusion cloning (Clontech, Mountain View, USA). Plasmids were transformed into C41 (DE3) cells harbouring a co-expression plasmid, pCDF-Duet, expressing lambda phosphatase and human CDC37 under an IPTG inducible promoter. Transformed colonies were selected on agar plated containing terrific broth, 10 mM Glucose, 100 µg/mL kanamycin and 50 µg/mL spectinomycin overnight at 37 °C. Colonies were inoculated into liquid media containing the same components as the solid media and grown in baffled flasks to an optical density (OD^{600}) of 0.8 to 1.2. Cultures were cooled to 15 °C for 1 hour and then induced through the addition of 0.1 mM IPTG (for pOPINS) or 1 mM rhamnose and 0.1 mM IPTG (for pJ821) for 16 hours. Cells were pelleted and stored at -20 °C until required.

Cells were lysed as described in (Bunney et al., 2012) using a chemical and enzymatic lysis method. The clarified lysates were loaded onto a 5 ml HisTrap column (GE Healthcare, Amersham, UK) within an Akta Explorer 10 (GE Healthcare, Amersham, UK). Proteins were washed with His Buffer A (25 mM Tris.Cl, 500 mM NaCl, 40 mM Imidazole, 1 mM TCEP, pH 8.0) and eluted with a 20-column volume gradient to His Buffer B (25 mM Tris.Cl, 500 mM NaCl, 500 mM Imidazole, 1 mM TCEP, pH 8.0). Eluted fractions containing protein were pooled and 100 µL of 10mg/ml Ulp1 protease was added per 20 mL of eluted recombinant protein. Removal of the purification tag progressed overnight in Dialysis Buffer (25 mM Tris.Cl, 1 mM TCEP, pH 8.0). Cleaved proteins were passed again over a HisTrap column using Dialysis Buffer as loading and wash buffer. Proteins that did not bind to the column were collected and subsequently injected on a 5 ml

HiTrap Q (GE Healthcare, Amersham, UK) equilibrated in Q Buffer A (25 mM Tris.Cl, 20 mM NaCl, 1 mM TCEP, pH 8.0). Proteins were eluted over 20 column volumes to 50% of Q Buffer B (25 mM Tris.Cl, 1 M NaCl, 1 mM TCEP, pH 8.0) and eluted proteins collected in 10 ml fractions. Those fractions containing FGFR kinase domains were pooled and concentrated in Vivaspin (Vivaproducts, Littleton, USA) concentrating units. Proteins were stored between 5 and 20 mg/mL, after snap freezing in liquid N_2, at -80 °C.

Preparation of Phosphorylated FGFR proteins

In certain assays a phosphorylated form of the various FGFR proteins was required. Production of phosphorylated protein was carried out as outlined in (Bunney et al., 2012) and (Furdui et al., 2006). Briefly, FGFR1 kinase domain constructs were expressed in C41 (DE3) cells with no accessory plasmid present but otherwise the same procedure as described above was followed. Yields of kinase domain were on average a tenth of those expressed with the accessory plasmid. The expressed protein consisted of a mixture of several phosphorylation states. These were separated using a 1 ml Resource Q column (GE Healthcare, Amersham, UK), the Q Buffers and with 400 column volumes to 50 % of Q Buffer B. This method generated a chromatogram with four discernable peaks, representing the 0p, 1p, 2p and 3p forms. The individual peaks were concentrated separately and stored as outlined above.

Phasing, refinement and structure validation

The initial phases for FGFR1-2c Apo data were calculated by the molecular replacement method (MR) (Rossmann, 1990) using FGFR1-2c structure (PDB: 2FGI (Mohammadi et al., 1998)) as the starting model in Phaser (McCoy et al., 2007) from CCP4 software suite (Winn et al., 2011). The molecular replacement model from Phaser (McCoy et al., 2007) was refined using Phenix software suite (Adams et al., 2010). Initial phases for FGFR1-2c DOV and FGFR1-2c^{V561M} DOV were obtained by MR using refined FGFR1-2c Apo structure in Phaser (McCoy et al., 2007). The structures of FGFR1-2c DOV and FGFR1-2c^{V561M} DOV were refined using Refmac5 (Vagin et al., 2004) and Phenix (Adams et al., 2010) software suites respectively. Please refer to Supplemental Table S5 for X-ray data refinement statistics. The ligand TKI258 was modelled and fitted into the electron density maps of FGFR1-2c DOV and FGFR1-2cV561M and refined followed by addition of solvent molecules. A subset of 5 % of reflections were kept aside for Rfree calculation. The refined structures were validated using validation tools in Phenix (McCoy et al., 2007) and online server Molprobity (Chen et al., 2010). The refined and validated structures of

FGFR1-2c Apo, FGFR1-2c DOV and FGFR1-2c^{V561M} DOV were submitted to PDB and their PDB codes were 4UWY, 4UWZ and 4UX0 respectively.

Isothermal Titration Calorimetry

Heats of interaction were measured on a VP-ITC system (Microcal) with a cell volume of 1.458 ml. Protein was dialysed for 16 hours in ITC buffer (50 mM HEPES.NaOH, 150 mM NaCl, 1 mM TCEP, pH 7.5) at 4 °C, in the case of experiments performed with Dovitinib, 10 % (v/v) DMSO was included in the buffer. Proteins and inhibitors were quantified using a nanodrop and theoretical extinction coefficients calculated from protein parameters (http://web.expasy.org/protparam) or supplied by Selleck Chemicals, in the case of inhibitors. FGFR kinase domain proteins were loaded in the sample cell at between 10 and 25 μM and titrated with either PD173074 (156 μM) or Dovitinib (300 μM) in the syringe. The titrations were performed while samples were being stirred at 260 r.p.m. at 20 °C. A total of 30 injections was carried out with 7.5 μl injected each time (except the first injection when 2.5 μl was injected) and a 4 min interval between each injection to allow the baseline to stabilize. The data were fitted with a single site model to calculate the number of binding sites (n), the binding constant (K_a), the change in enthalpy (ΔH) and change in entropy (ΔS) using Origin software (Microcal).

Computational Docking

Computational protein-ligand docking was used to gain insight into the binding mode of Dovitinib to the FGFR1 kinase domain. To increase confidence in the docking results, the best poses were analysed further using molecular dynamics simulation.

Computational docking was performed using both SwissDock (Grosdidier et al., 2011b) and AutoDock Vina (v.1.1.2) (Trott and Olson, 2010). SwissDock is a web-based docking server based on the EADock DSS method (Grosdidier et al., 2011a). AutoDock Vina docks ligands by optimising a scoring function using a stochastic global search.

Docking runs were carried out using the kinase domain of FGFR1 from PDB ID: 2FGI (Mohammadi et al., 1998). Docking preparation was performed with UCSF Chimera v1.9 (build 39798) (Pettersen et al., 2004). Chain B and the complexed inhibitor PD173074 were deleted. The ‘Dock Prep’ module options selected were: delete solvent; delete non-complexed ions; alternative locations - keep highest occupancy; if present change MSE to MET, 5BU to UMP, UMS to UMP, CSL to CMP; incomplete side chains replaced using Dunbrack rotamer library (Dunbrack, 2002); add hydrogens; add charges. Protonation

states were assigned for histidine, glutamic acid, aspartic acid, lysine and cysteine using residue-names and by considering H-bonds. Charges were assigned using AMBER ff12SB (Case et al., 2012).

Ligands from the Zinc database (Irwin and Shoichet, 2005) were prepared automatically by the SwissDock server for docking. However, AutoDock Vina requires ligands in a modified PDB format ('pdbqt') that includes partial charges. For Vina, Dovitinib was downloaded from Zinc in mol2 format (http://zinc.docking.org/substance/ 3816310) and either (i) prepared with and then exported from SwissDock or (ii) prepared with AutoDock Tools (ADT) (Morris et al., 2009).

For docking with SwissDock. The prepared protein structure was uploaded to the SwissDock server (http://www.swissdock.ch/docking). Dovitinib was chosen via a direct Zinc database accession search for '3816310' and docking using the 'Accurate' option. Docking search space was not constrained to the active site, so only ligand clusters in the site were chosen from the total of 256 poses in 37 separate clusters. In total, 3 clusters of poses were chosen with the following reported $\triangle G$ values (top scoring pose in each cluster and the mean/standard deviation, μ, for each cluster):

$Clust_3$: $\triangle G_{top} = -8.63$ kcal mol^{-1}, $\mu = -8.25 \pm 0.77$ kcal mol^{-1}

$Clust_6$: $\triangle G_{top} = -8.43$ kcal mol^{-1}, $\mu = -8.43 \pm 0.00$ kcal mol^{-1}

$Clust_{14}$: $\triangle G_{top} = -8.45$ kcal mol^{-1}, $\mu = -7.88 \pm 0.54$ kcal mol^{-1}.

For docking with AutoDock Vina. Following preparation of the protein, ADT, operating within the PyRx software suite (Wolf, 2009), was used to generate the 'pdbqt' format required by Vina. The docking search space was set by finding the mean of all atoms in PDB ID: 2FGI within 10 Å of the structure's ligand PD173074; a cube of side 20 Å centered on (4.88, 3.30, 17.62) was used for all Vina docking calculations, with parameter 'exhaustiveness' set to 8. Vina reported the top 9 poses with the following estimates of $\triangle G$, using ligands prepared using either ADT or SwissDock:

$Poses_{ADT\_prep}$: $\triangle G_{top} = -8.2$ kcal mol^{-1}, $\mu = -7.3 \pm 0.5$ kcal mol^{-1}

$Poses_{SwissDock\_prep}$: $\triangle G_{top} = -8.0$ kcal mol^{-1}, $\mu = -7.4 \pm 0.4$ kcal mol^{-1}

Molecular Models

The initial coordinates for the FGFR1 bound with PD173074 were taken from the x-ray crystal structure (PDB id: 2FGI) (Mohammadi et al., 1998). The V561M and Y563C mutant complexes were obtained by substituting the valine 561 or tyrosine 563 residues in the wild-type structure with methionine or cysteine, respectively. Gaussian 03 (Frisch et al., 2004) was used to determine electrostatic potential of PD173074 at the Hartree-Fock level with 6-31G** basis functions. The restrained electrostatic potential (RESP) module in the Amber package (Case et al., 2005) was used to calculate the partial charges, and the general Amber force field (GAFF) was used to assign the force field parameters for the inhibitor. Protein parameters were taken from the standard Amber force field (ff99SBildn) (Lindorff-Larsen et al., 2010). The complexes were solvated in orthorhombic water boxes that extended at least 14Å from the complexes.

Thermodynamic Integration

All simulations were performed using the NAMD2.9 package (Phillips et al., 2005) in the NPT ensemble with a temperature of 300K and a pressure of 1 atm. The thermodynamic integrations (TI) were performed in two directions: from wild type to mutant (forward) and vice versa (reverse). The difference, often called “hysteresis”, of TI calculations between the two directions provides an indication as to whether the sampling is adequate. The simulations were run with an alchemical parameter λ=0.05, 0.1, 0.2, …, 0.8, 0.9, 0.95. A soft-core potential was used for van der Waals interactions involving mutant atoms. The electrostatic interactions were linearly scaled down for annihilating atoms at a rate of $2*(1-\lambda)$ for λ=0.05-0.5, and up at a rate of $2*(\lambda-0.5)$ for λ=0.5-0.95. The separate scaling method avoids the so-called “end-point catastrophe” by decoupling the electrostatic interaction completely before the mutant atoms are fully annihilated, and coupling after the growing atoms have appeared. 11 nanosecond MD simulation was performed for each λ window. The last 10ns of the simulations were collected for TI calculations. Three replicas of each alchemical mutation were performed to improve sampling statistics. The simulations were performed on 96 or 192 cores on ARCHER (Cray XC30), the UK national supercomputer based in Edinburgh.

Molecular Dynamics calculations

The MD simulations were performed using GROMACS 4.5 (Hess et al., 2008) with the PLUMED plug-in (Bonomi et al., 2009) for parallel tempering metadynamics calculations and the Charmm22* force field (Bjelkmar et al., 2010). The wild type and the mutant FGFR systems were solvated with TIP3P water molecules (Mahoney and Jorgensen, 2000) and enclosed in a dodecahedral box with periodic boundary conditions. The van

der Waals interactions were cut-off at 1.0 nm and shifted to zero; the long-range electrostatic interactions were calculated by the particle mesh Ewald algorithm (Essmann et al., 1995), with mesh spaced 0.12 nm, and shifted to zero above 1.0 nm to make the potential the integral of the force. The system evolves in the canonical ensemble, coupled with a velocity-rescale thermostat (Bussi et al., 2007) and a time step of 2 fs. Each solvated system was prepared as detailed in SI text.

Enhanced Sampling

Parallel Tempering Metadynamics (PT-metaD) (Bussi et al., 2006) was performed for WT and V561M FGFR using 20 replicas at increasing temperatures (range: 298.0 K - 337.5 K). All 20 replicas are subject to the well-tempered metadynamics prescription in which a Gaussian is deposited in the collective variable space every 2 ps with height W=W0*exp(-V(s,t)/(f-1)T), where W0=10 kJ/mol is the initial height, T is the temperature of the replica, f = 10 is the bias factor, and V(s,t) is the bias potential at time t and CV value s. The following two collective variables (cv) are used: the distance in contact map space to the inactive A-loop conformation CV1(R)=… and the distance in contact map space to the active conformation CV2(R)=...

The widths of the Gaussians in the two CV dimensions are σ1 = 0.5 and σ2 = 0.5. Note that the CVs are adimensional.

Analysis

The free energy surfaces as a function of the two CVs are obtained by integrating the deposited bias during the simulation, as required by the metadynamics algorithm. To obtain a representative structure of each free energy basin, a clustering of the set of structures falling within a small (CV1, CV2) area surrounding each basin has been performed. The single-linkage clustering algorithm of g_cluster program from the GROMACS package has been used with the RMSD on the CA atoms as the distance with a cutoff of 0.2 nm. In the most populated cluster of each basin, the central structure, i.e., the structure with smallest distance to all of the other members of the cluster 1 has been picked as representative of the basin.

Nuclear Magnetic Resonance

A Bruker Avance III (800 MHz) spectrometer equipped with a cryogenically cooled triple resonance probe with a z-axis pulse field gradient coiled was used for data collection. Spectra were recorded at 25 ˚C or 30 ˚C in NMR buffer (50 mM PIPES.NaOH, 25 mM NaCl, 5 mM TCEP, 1 mm EDTA, pH 7.0) and 10% D_2O. The ^{2}H,^{15}N,^{13}C-labelled FGFR3 kinase sample was used at a concentration of 210 μM. For backbone-resonance assignment, a full set of standard backbone resonance assignment experiments (TROSY versions with ^{2}H decoupling) was recorded for the PD173075 (1:1.1) - bound triple-labelled sample. For apo-FGFR3 kinase and for TKI258-bound FGFR3 kinase, only HNCA and HNCO experiments were performed. NMR raw data were processed with NMRPipe and NMRDraw (Delaglio et al., 1995). Spectra were analysed with CcpNmr Analysis (Vranken et al., 2005). Backbone assignment of apo-FGFR1 kinase, as reported elsewhere (Vajpai et al., 2014), aided the assignment of apo-FGFR3 kinase. Chemical shift perturbation was calculated from ^{1}H and ^{15}N chemical shift differences by multiplying the ^{15}N shift differences using 0.14 as a scaling factor for ^{15}N shift changes (Williamson, 2013). Due to the still incomplete state of FGFR3 kinase backbone resonance assignment, the chemical shift analysis was only performed for 30% of the residues (not including proline residues) in FGFR kinase, ensuring that major chemical shift differences were taken into account. For visualization purposes, the chemical shift perturbation data were encoded as occupancy factors in the respective pdb file. These were projected on the structure using VMD 1.9.1 (Humphrey, 1996). Current, overall state of assignment of FGFR3 KD shows that it is closely related to previously published assignment for FGFR1 (Vajpai et al., 2014) and that this was sufficient for describing shifts in FGFR3 occurring upon the drug binding.

Protein extraction and immunoprecipitation

Cultured cells were lysed in RIPAE buffer (1% (v/v) Triton X-100, 1 mM EDTA, 0.1% (w/v) SDS, 0.5% (w/v) sodium deoxycholate, 10% (v/v) glycerol in PBS) with protease inhibitor cocktail (Sigma) and phosphatase inhibitor cocktail 2 (Sigma). Lysates were cleared by centrifugation for 15 min at 12,700 x g at 4°C. Protein concentrations were determined using the Bio-Rad Protein Assay Dye Reagent (Bio-Rad Laboratories, Hemel-Hempstead, UK). For immmunoprecipitation protein was incubated with antibodies to FGFR3 extracellular domain (F3922, Sigma) overnight at 4°C, followed by the addition of protein A sepharose beads (Amersham Biosciences) for 4 hr. The beads were washed twice in PBS, resuspended in 2xSDS loading buffer with beta-mercaptoethanol, boiled for 5 mins and run on precast 7.5% SDS-polyacrylamide gels (BioRad). Proteins were transferred to nitrocellulose membrane using the trans-blot turbo transfer system (BioRad).

Primary antibodies used were 4G10 antiphosphotyrosine (1:1000 in 3% (w/v) BSA overnight at 4°C) (Millipore, Watford, UK) and FGFR3 B9 (1:1000 in 2% (w/v) non-fat dried milk (NFDM) 1 hr at room temperature (RT) (Santa Cruz Biotechnology, Santa Cruz, CA, USA). Proteins were visualised with chemiluminescence using horseradish peroxidase conjugated secondary antibodies, and Luminata Forte Western HRP Substrate (Millipore). Blots were stripped in 50 mM Tris pH 7.5, 10 M urea at 55°C for 50 mins before re-probing.

Cell Culture

NIH-3T3 cells were cultured in Dulbecco's Modified Eagles Medium (Sigma) with 10% FBS (Invitrogen) and 2mM L-glutamine, at 37°C in 10% CO_2.

Expression vectors and transduction into NIH-3T3

Wild Type (IIIb), K652E and S249C mutant FGFR3 constructs in retroviral expression vector pFB (Stratagene) containing a hygromycin resistance casette were as described (di Martino et al., 2009). Construct with RT112FUS (FGFR3:TACC3 fusion from RT112 cell line) was as described (Williams et al., 2013).

The expression vectors were transfected into Phoenix A cells using TransIT-293 transfection reagent (Mirus). NIH-3T3 cells were incubated with retroviral supernatant containing 8 µg/ml polybrene for 6 hours and selected with 200 µg/ml hygromycin 72h after transduction.

Growth in soft agar

Transduced 3T3 cells were cultured in medium containing 0.4% agarose, on a base of medium containing 0.8% agarose (BD baculogold or Gibco). They were seeded in triplicate at $5x10^{3}$ cells per well in 6-well plates and fed weekly. Colonies bigger than 100µm were counted on day 14, after staining with 8mM p-iodonitrotetrazolium violet (Sigma). Results are from at least 3 independent assays.

Cell number

Cells were seeded in triplicate at $1x10^5$ cells per well in 6-well plates, fed every 2-3 days and trypsinised and counted after 7 days, using a coulter counter (Beckman). Results are from 3 independent assays.

Cell Viability Assay

The amount of viable cells following culture with kinase inhibitors was measured using the CellTiter-Blue Cell Viability Assay (Promega). Cells were plated in triplicate in a 96-well plate at $2x10^3$ cells/well and left to attach overnight before the addition of fresh medium with inhibitor at the indicated concentration. After 3 days with the inhibitor, 20 µl of cell titer blue reagent was added for 2 hours, and fluorescence measured with excitation at 540 nm and emission at 590 nm, using a Mithras LB940 plate reader (Berthold technologies). Background signal was removed by subtracting the fluorescence from cell-free wells. Results are from 3 independent assays.

Supplemental Table S1: Inhibition constants of FGFR kinase domains and their mutants.

Protein	State	Inhibitor	K_i
$FGFR1^{WT}$	Apo	PD173074	40.0 ± 8.6
		Dovitinib	111.6 ± 13.4
		AZD4547	6.1 ± 2.2
		Ponatinib	62.7 ± 11.3
	Phospho	PD173074	10.4 ± 1.1
		Dovitinib	121.2 ± 5.4
		AZD4547	2.7 ± 0.6
		Ponatinib	24.6 ± 1.4
$FGFR1^{V561M}$	Apo	PD173074	1580 ± 110
		Dovitinib	34.6 ± 2.6
		AZD4547	82.1 ± 4.5
		Ponatinib	92.4 ± 4.6
	Phospho	PD173074	≈ 9000
		Dovitinib	142.0 ± 15.7
		AZD4547	131.8 ± 12.4
		Ponatinib	301.2 ± 52.3
$FGFR1^{Y563C}$	Apo	PD173074	104.2 ± 10.4
		Dovitinib	801.2 ± 85.6
		AZD4547	45.2 ± 10.3
		Ponatinib	162.0 ± 26.1
$FGFR3^{WT}$	Apo	PD173074	25.7 ± 6.7
		Dovitinib	65.4 ± 14.6
		AZD4547	4.4 ± 2.4
		Ponatinib	94.5 ± 12.2
$FGFR3^{V555M}$	Apo	PD173074	877.4 ± 90.6
		Dovitinib	12.9 ± 3.8
		AZD4547	82.5 ± 10.9
		Ponatinib	184.5 ± 18.9

Supplemental Table S2: Kinetic Parameters of FGFR kinase domains and their mutants.

Protein	State	$K_m^{(ATP)}$ (μM)	$k_{cat}^{(ATP)}$ (s^{-1})	$k_{cat}/K_m^{(ATP)}$ ($\mu M^{-1}s^{-1}$)
$FGFR1^{WT}$	Apo	184.4 ± 14.7	0.043	2.33E-4
	Phospho	70.5 ± 6.9	2.035	2.89E-2
$FGFR1^{V561M}$	Apo	121.4 ± 14.0	0.170	1.40E-3
	Phospho	44.17 ± 3.7	1.357	3.07E-2
$FGFR1^{Y563C}$	Apo	367.2 ± 43.5	0.032	8.71E-5
$FGFR3^{WT}$	Apo	326.8 ± 34.7	0.062	1.90E-4
$FGFR3^{V555M}$	Apo	111.7 ± 10.6	0.143	1.28E-3
$FGFR3^{K650E}$	Apo	81.7 ± 6.7	0.441	5.40E-3

Supplemental Table S3: Thermodynamic Quantities for the Binding of FGFR kinase domains and their mutants to various ATP-competitive inhibitors.

Protein	Ligand	n	K_d (nM)	ΔH (kcal mol^{-1})	-TΔS (kcal mol^{-1})	ΔG (kcal mol^{-1})
FGFR3^{WT}	PD173074	0.89 ± 0.002	2.64 ± 1.23	-11.4 ± 0.07	-0.1 ± 0.32	-11.5 ± 0.25
FGFR1^{WT}	PD173074	0.91 ± 0.0007	2.81 ± 0.36	-7.7 ± 0.01	-3.7 ± 0.1	-11.4 ± 0.09
	Dovitinib	1.12 ± 0.007	185 ± 14	-4.5 ± 0.04	-4.5 ± 0.72	-9.0 ± 0.68
FGFR1^{V561M}	PD173074	0.80 ± 0.006	307 ± 35	-3.4 ± 0.04	-5.3 ± 0.14	-8.7 ± 0.10
	Dovitinib	0.91 ± 0.006	64.5 ± 3.1	-6.9± 0.07	-2.7 ± 0.53	-9.6 ± 0.46
FGFR1^{Y563C}	PD173074	0.66 ± 0.002	14.8 ± 1.47	-6.4 ± 0.03	-4.1 ± 0.14	-10.5 ± 0.11

Supplemental Table S4: Binding Free Energy Differences of PD173074 for the FGFR^{Y563C} mutant Calculated from Thermodynamic Integration based on Molecular Dynamic Simulations and Determined Experimentally by ITC (kcal/mol).

simulation	Energy	rep1	rep2	rep3	rep4	rep5	average	$\Delta\Delta G_{theor}$	$\Delta\Delta G^{avg}_{theor}$	$\Delta\Delta G_{exp}$
forward	$\Delta G^{alch}_{protein}$	-10.19	-8.67	-10.47	-10.25	-10.20	-9.96	1.10	0.89 (0.21)	0.97 (0.20)
	$\Delta G^{alch}_{complex}$	-11.03	-11.01	-10.87	-11.18	-11.22	-11.06			
reverse	$\Delta G^{alch}_{protein}$	-9.74	-9.94	-10.43	-10.66	-10.55	-10.26	0.68		
	$\Delta G^{alch}_{complex}$	-11.53	-10.01	-11.53	-10.56	-11.09	-10.94			

Supplemental Table S5: X-ray data collection and refinement statistics.

Space group	C 2	C 2	C 2
Cell dimensions	*a* = 211.8 Å; *b* = 49.8 Å; *c* = 66.1 Å; *α* = *γ* = 90°; *β* = 107.4°	*a* = 208.7 Å; *b* = 57.6 Å; *c* = 65.6 Å; *α* = *γ* = 90°; *β* = *107.4°*	*a* = 208.6 Å; *b* = 57.8 Å; *c* = 65.6 Å; *α* = *γ* = 90°; *β* = 107.4°
Resolution range (Å)	27.81 – 2.30	43.26 – 1.96	28.92 – 1.96
*R*symm[a] (outer shell)	0.071 (0.476)	0.066 (0.482)	0.050 (0.318)
I/σ*I* (outer shell)	12.8 (2.5)	12.3 (2.6)	13.0 (1.9)
Completeness (outer shell) %	91.1 (50.0)	96.0 (95.6)	83.2 (29.4)
Total number of reflections	127,053	228,641	169.979
Number of unique reflections	26,800	51,344	45,034
Redundancy (outer shell)	4.7 (4.4)	4.5 (4.2)	3.8 (2.0
Wilson *B*-factor ($Å^2$)	38.65	34.2	31.05
R_{cryst}[b]/R_{free}[c]	0.211/0.267	0.214/0.253	0.199/0.255
Average B-factor ($Å^2$)			
Overall	52.44	47.95	50.85
Protein (chain A, B)	45.95, 51.07	42.83, 46.88	47.21, 51.72
Chloride ion (3)	59.67	58.65	59.46
Solvent	45.50	47.75	47.42
Ligand	60.0 (PEG)	44.2, 47.4 (Dovitinib)	45.7, 53.6 (Dovitinib)
RMS deviation			
Bond length (Å)	0.005	0.007	0.010
Bond angle (°)	0.927	1.182	1.372
Ramachandran plot statistics			
Favoured (%)	97.14	97.34	97.72
Less favoured (%)	2.86	2.66	2.28
PDB ID	**4UWY**	**4UWZ**	**4UX0**

[a]R_{symm} = Σ*h* Σ*i* |*I*(*h*) − *Ii*(*h*)|/Σ*h* Σ*iIi*(*h*), where *Ii*(*h*) and *I*(*h*) are the *i*th and the mean measurements of the intensity of reflection *h*, respectively.

[b]R_{cryst} = Σ*h* |*F*o − *F*c|/Σ*hF*o, where *F*o and *F*c are the observed and calculated structure factor amplitudes of reflection *h*, respectively.

[c]R_{free} is equal to R_{cryst} for a randomly selected 5.0% subset of reflections not used in the refinement.

Supplemental Table S6: Binding of TKI258 (dovitinib, DOV) to FGFR1 KD

A) *Hydrogen bonds and hydrophobic interactions between the ligand and FGFR1.*

	Ligand atom	***Protein atom***	***Distance (Å)***
Hydrogen bonds	DOV *N7*	E562 *O*	3.09
	DOV *O28*	A564 *N*	2.40
	DOV *O28*	A564 *O*	3.10
	DOV *N12*	A564 *O*	2.64
	DOV *N20*	S565 *O*	3.38
Hydrophobic interaction	L484, V492, A512, K514#, I545, Y563, G567, L630, A640		

#K514 exhibits hydrophobic interaction with the ligand only in the native FGFR1-2c. In FGFR1^{V561M} DOV complex, the C^{ε} atom of M561 pushes C^{ε} atom of K514 away and thus exhibits a weak hydrophobic interaction.

B) *Accessible surface area calculation at the ligand-binding pocket*

PDB:Ligand	***Total area (Ligand) Å²***		***Total contact area (Ligand) Å²***	
	Chain **A**	Chain **B**	Chain **A**	Chain **B**
4UWY	15071.9	14265.2	4433.3	4193.4
4UWZ:DOV$	15176.1 (195.9)	14372.3 (197.6)	4458.3 (59.3)	4225.7 (59.7)
4UX0:DOV$	15049.3 (200.3)	14937.7 (200.6)	4426.8 (60.5)	4386.1 (60.5)
2FGI:PD173074#	13349.6 (253.2)	12908.1 (224.0)	3936.3 (77.6)	3803.7 (68.8)
3GQI:ACP	16337.4 (107.6)	-	4863.2 (30.1)	-
2PY3:ACP	12309.6 (165.8)	12330.7 (166.1)	3627.1 (47.1)	3636.2 (47.3)
2PVF:ACP	14367.4 (101.8)	-	4222.3 (28.8)	-

#The difference in the total and contact area for the ligand is because of absence of amino acid residues from 486-490 (P-loop) in chain A.

$In PDBs 4UWZ (except for chain B) and 4UX0 amino acid residues 487-489 (P-loop) could not be modelled. The differences in either total area and total contact area are due to the number of atoms included in the surface area calculation that do not affect the ligand binding pocket.

C) *Root mean square deviation (RMSD) of FGFR1 structures.*

**PDB ID**	***4UWY***	***4UWZ***	***4UX0***	***3GQI***	***3GQL***	***2FGI***	***2PY3***	***2PVF***
4UWY (297)	-							
4UWZ (295)	1.01 (286)	-						
4UX0 (294)	1.02 (287)	0.36 (293)	-					
3GQI (304)	2.13 (283)	1.93 (279)	1.83 (279)	-				
3GQL (287)	0.85 (271)	1.00 (275)	0.91 (274)	2.12 (275)	-			
2FGI (274)	0.99 (274)	0.35 (274)	0.35 (274)	1.57 (266)	0.81 (264)	-		
2PY3 (282)	2.00 (274)	1.90 (273)	2.03 (274)	1.42 (279)	2.18 (269)	1.99 (266)	-	
2PVF (285)	1.60 (274)	1.54 (271)	1.56 (271)	1.22 (283)	2.01 (269)	1.74 (266)	1.46 (275)	-

The total number of residues and total number of aligned residues per molecule are given in braces next to their PDB ID and RMSD values respectively. The RMSD are in Å units. RMSD values are calculated using the program TM-align [14].

Supplemental Table S7: Free energy changes (kcal/mol) calculated by thermodynamic integration of apo-FGFR1 upon gatekeeper mutation V561M at the active and inactive conformations. $\Delta G_{conformation} = G^{WT}_{conformation} - G^{Mut}_{conformation}$

Energy	WT → Mut					Mut → WT					avg-total
	rep1	rep2	rep3	rep4	avg	rep1	rep2	rep3	rep4	avg	
$\Delta G_{inactive}$	1.03	0.46	1.08	0.60	0.79	0.73	0.51	0.77	1.31	0.83	0.81±0.02
ΔG_{active}	0.89	2.40	1.09	1.35	1.43	1.69	1.09	1.07	0.94	1.20	1.32±0.12
ΔΔG					-0.64					-0.37	-0.51±0.14

Supplemental References

Adams, P. D., Afonine, P. V., Bunkoczi, G., Chen, V. B., Davis, I. W., Echols, N., Headd, J. J., Hung, L. W., Kapral, G. J., Grosse-Kunstleve, R. W., *et al.* (2010). PHENIX: a comprehensive Python-based system for macromolecular structure solution. Acta crystallographica Section D, Biological crystallography *66*, 213-221.

Bjelkmar, P., Larsson, P., Cuendet, M. A., Hess, B., and Lindahl, E. (2010). Implementation of the CHARMM Force Field in GROMACS: Analysis of Protein Stability Effects from Correction Maps, Virtual Interaction Sites, and Water Models. J Chem Theory Comput *6*, 459-466.

Bonomi, M., Branduardi, D., Bussi, G., Camilloni, C., Provasi, D., Raiteri, P., Donadio, D., Marinelli, F., Pietrucci, F., Broglia, R. A., and Parrinello, M. (2009). PLUMED: A portable plugin for free-energy calculations with molecular dynamics. Comput Phys Commun *180*, 1961-1972.

Bunney, T. D., Esposito, D., Mas-Droux, C., Lamber, E., Baxendale, R. W., Martins, M., Cole, A., Svergun, D., Driscoll, P. C., and Katan, M. (2012). Structural and functional integration of the PLCgamma interaction domains critical for regulatory mechanisms and signaling deregulation. Structure *20*, 2062-2075.

Bussi, G., Donadio, D., and Parrinello, M. (2007). Canonical sampling through velocity rescaling. J Chem Phys *126*.

Bussi, G., Gervasio, F. L., Laio, A., and Parrinello, M. (2006). Free-energy landscape for beta hairpin folding from combined parallel tempering and metadynamics. J Am Chem Soc *128*, 13435-13441.

Case, D. A., Cheatham, T. E., 3rd, Darden, T., Gohlke, H., Luo, R., Merz, K. M., Jr., Onufriev, A., Simmerling, C., Wang, B., and Woods, R. J. (2005). The Amber biomolecular simulation programs. J Comput Chem *26*, 1668-1688.

Case, D. A., Darden, T., Cheatham, T. E., Simmerling, C. L., Wang, J., Duke, R. E., Luo, R., Walker, R. C., Zhang, W., Merz, K. M., and al., e. (2012). AMBER 12. University of California, San Francisco *1*, 3.

Chen, V. B., Arendall, W. B., 3rd, Headd, J. J., Keedy, D. A., Immormino, R. M., Kapral, G. J., Murray, L. W., Richardson, J. S., and Richardson, D. C. (2010). MolProbity: all-

atom structure validation for macromolecular crystallography. Acta crystallographica Section D, Biological crystallography *66*, 12-21.

Delaglio, F., Grzesiek, S., Vuister, G. W., Zhu, G., Pfeifer, J., and Bax, A. (1995). NMRPipe: a multidimensional spectral processing system based on UNIX pipes. Journal of biomolecular NMR *6*, 277-293.

di Martino, E., L'Hote, C. G., Kennedy, W., Tomlinson, D. C., and Knowles, M. A. (2009). Mutant fibroblast growth factor receptor 3 induces intracellular signaling and cellular transformation in a cell type- and mutation-specific manner. Oncogene *28*, 4306-4316.

Dunbrack, R. L. (2002). Rotamer libraries in the 21(st) century. Curr Opin Struc Biol *12*, 431-440.

Essmann, U., Perera, L., Berkowitz, M. L., Darden, T., Lee, H., and Pedersen, L. G. (1995). A Smooth Particle Mesh Ewald Method. J Chem Phys *103*, 8577-8593.

Frisch, M. J., Trucks, G. W., Schlegel, H. B., Scuseria, G. E., Robb, M. A., Cheeseman, J. R., Montgomery, J. A., Vreven, T., Kudin, K. N., and Burant, J. C. (2004). Guassian 03, Revision B.03. In, (Guassian, Inc, Wallingford, CT 06492 USA).

Furdui, C. M., Lew, E. D., Schlessinger, J., and Anderson, K. S. (2006). Autophosphorylation of FGFR1 kinase is mediated by a sequential and precisely ordered reaction. Molecular cell *21*, 711-717.

Grosdidier, A., Zoete, V., and Michielin, O. (2011a). Fast Docking Using the CHARMM Force Field with EADock DSS. J Comput Chem *32*, 2149-2159.

Grosdidier, A., Zoete, V., and Michielin, O. (2011b). SwissDock, a protein-small molecule docking web service based on EADock DSS. Nucleic Acids Res *39*, W270-W277.

Hess, B., Kutzner, C., van der Spoel, D., and Lindahl, E. (2008). GROMACS 4: Algorithms for highly efficient, load-balanced, and scalable molecular simulation. J Chem Theory Comput *4*, 435-447.

Humphrey, W., Dalke, A. and Schulten, K., "VMD - Visual Molecular Dynamics", J. Molec. Graphics, 1996, vol. 14, pp. 33-38.

Irwin, J. J., and Shoichet, B. K. (2005). ZINC - A free database of commercially available compounds for virtual screening. J Chem Inf Model *45*, 177-182.

Lindorff-Larsen, K., Piana, S., Palmo, K., Maragakis, P., Klepeis, J. L., Dror, R. O., and Shaw, D. E. (2010). Improved side-chain torsion potentials for the Amber ff99SB protein force field. Proteins *78*, 1950-1958.

Mahoney, M. W., and Jorgensen, W. L. (2000). A five-site model for liquid water and the reproduction of the density anomaly by rigid, nonpolarizable potential functions. J Chem Phys *112*, 8910-8922.

McCoy, A. J., Grosse-Kunstleve, R. W., Adams, P. D., Winn, M. D., Storoni, L. C., and Read, R. J. (2007). Phaser crystallographic software. Journal of applied crystallography *40*, 658-674.

Mohammadi, M., Froum, S., Hamby, J. M., Schroeder, M. C., Panek, R. L., Lu, G. H., Eliseenkova, A. V., Green, D., Schlessinger, J., and Hubbard, S. R. (1998). Crystal structure of an angiogenesis inhibitor bound to the FGF receptor tyrosine kinase domain. The EMBO journal *17*, 5896-5904.

Morris, G. M., Huey, R., Lindstrom, W., Sanner, M. F., Belew, R. K., Goodsell, D. S., and Olson, A. J. (2009). AutoDock4 and AutoDockTools4: Automated Docking with Selective Receptor Flexibility. J Comput Chem *30*, 2785-2791.

Pettersen, E. F., Goddard, T. D., Huang, C. C., Couch, G. S., Greenblatt, D. M., Meng, E. C., and Ferrin, T. E. (2004). UCSF chimera - A visualization system for exploratory research and analysis. J Comput Chem *25*, 1605-1612.

Phillips, J. C., Braun, R., Wang, W., Gumbart, J., Tajkhorshid, E., Villa, E., Chipot, C., Skeel, R. D., Kale, L., and Schulten, K. (2005). Scalable molecular dynamics with NAMD. J Comput Chem *26*, 1781-1802.

Rossmann, M. G. (1990). The molecular replacement method. Acta crystallographica Section A, Foundations of crystallography *46 (Pt 2)*, 73-82.

Trott, O., and Olson, A. J. (2010). Software News and Update AutoDock Vina: Improving the Speed and Accuracy of Docking with a New Scoring Function, Efficient Optimization, and Multithreading. J Comput Chem *31*, 455-461.

Vagin, A. A., Steiner, R. A., Lebedev, A. A., Potterton, L., McNicholas, S., Long, F., and Murshudov, G. N. (2004). REFMAC5 dictionary: organization of prior chemical knowledge and guidelines for its use. Acta crystallographica Section D, Biological crystallography *60*, 2184-2195.

Vajpai, N., Schott, A. K., Vogtherr, M., and Breeze, A. L. (2014). NMR backbone assignments of the tyrosine kinase domain of human fibroblast growth factor receptor 1. Biomolecular NMR assignments *8*, 85-88.

Vranken, W. F., Boucher, W., Stevens, T. J., Fogh, R. H., Pajon, A., Llinas, M., Ulrich, E. L., Markley, J. L., Ionides, J., and Laue, E. D. (2005). The CCPN data model for NMR spectroscopy: development of a software pipeline. Proteins *59*, 687-696.

Williams, S. V., Hurst, C. D., and Knowles, M. A. (2013). Oncogenic FGFR3 gene fusions in bladder cancer. Human molecular genetics *22*, 795-803.

Williamson, M. P. (2013). Using chemical shift perturbation to characterise ligand binding. Progress in nuclear magnetic resonance spectroscopy *73*, 1-16.

Winn, M. D., Ballard, C. C., Cowtan, K. D., Dodson, E. J., Emsley, P., Evans, P. R., Keegan, R. M., Krissinel, E. B., Leslie, A. G., McCoy, A.*, et al.* (2011). Overview of the CCP4 suite and current developments. Acta crystallographica Section D, Biological crystallography *67*, 235-242.

Wolf, L. K. (2009). New software and websites for the chemical enterprise. Chem Eng News *87*, 31.

Supplemental Figure Legends

Supplemental Figure S1. Structural insights into TKI528 binding to FGFR1 and FGFR3 KD

A. Cartoon and ball-and-stick representation of FGFR1 KD bound to TKI258. TKI258 atoms are shown as pink spheres that approximates their van der Waals contact surface area. The residues in contact with TKI258 from both WT FGFR1 KD (left panel) and V561M FGFR1 KD (right panel) are shown as yellow spheres that approximates their van der Waals contact surface area. Coloured in black gatekeeper residue V/M561 and in blue are the residues corresponding to DFG motif. Below each of the contact surface area are LigPlot representations of TKI258 and its interacting residues.

B. Comparison of apo-FGFR3 kinase with TKI528-bound FGFR3 kinase by NMR.

Left panel: Chemical shift perturbation of the N and H backbone atoms, induced by TKI528 binding to FGFR3 kinase. These shift differences were projected on the homologous FGFR1 kinase / TKI528 crystal structure (colour scale shown in the figure; grey: no data). Residues that are very distant from the putative drug-binding site do not show large perturbations.

Right panel: FGFR3 kinase region with major chemical shift differences upon TKI528 binding in detail. The FGFR3 kinase residues with the 15 highest chemical shift perturbations upon TKI528 binding are shown in a side chain representation (green, yellow). According to the crystal structure of FGFR1 kinase / TKI528, 5 of these residues are potentially in in direct contact with the drug (green); other residues which are thought to be in direct contact with the drug have not been assigned. Other assigned residues with notable chemical shift perturbations are probably not in direct contact with the drug.

Supplemental Figure S2. Structural comparison of FGFR KDs

A. Comparison of dynamic loop regions in various FGFR KD molecules. The activation loop is coloured in red, P-loop in violet, hinge region in orange and catalytic loop in green. The close-up view of these regions are shown in various colours based on PDB codes: FGFR1, 4UWY (deep teal and marine), 4UWZ (limon and split pea), 4UX0 (deep teal and marine), 1FGK (raspberry), 3GQI (brown), 3GQL (salmon); FGFR2, 2PY3 (light orange), 2PVF (deep olive); FGFR3, 4K33 (chartreuse). For the activation loop the position of DFG motif and tyrosine residues are shown for both active and inactive enzyme. The residues labelled in the activation loop are for FGFR1 only.

B. Comparison of the hydrophobic (or regulatory) spine (R-spine), the catalytic spine (C-spine) and the molecular break residues in various FGFR KD molecules. WT FGFR1 KD is represented as a cartoon with an atomic surface around it. The secondary structural features are coloured differently.

Residues that belong to C-spine, R-spine and molecular break are shown as ball and stick model within respective boxes. Labelled in black are for FGFR1, brown for FGFR2 and green for FGFR3. For the activated states, colours are according PDB codes: FGFR1 (PDB: 3GQI) is coloured in orange, FGFR2 (PDB: 2PVF) in violet, molecular break mutant of FGFR2 E565G (PDB: 2PY3) in purple and auto activating FGFR3 mutant K650E (PDG: 4K33) in chartreuse. Residues in blue (R-spine), red (C-spine) and forest green (molecular break) represent the inactive conformations.

Supplemental Figure S3. Comparison of non-phospho and activated, phospho forms of FGFR1 KD and consequences for the TKI258 binding

Overall structural superposition of non-phospho WT FGFR1 KD/TKI258 (PDB: 4UWY) and activated and ACP bound WT FGFR1 KD (PDB: 3GQI) is shown in (**A**) and close ups for each structure, focusing on the ATP/TKI258 binding pocket in (**B**) and (**C**), respectively. The C-lobe of WT FGFR1 KD//TKI258 and ACP-bound FGFR1 KD are coloured in pale cyan and pale green respectively, while the N-lobes are coloured in orange and forest green, respectively. The activation loops for WT FGFR1 KD//TKI258 and ACP bound FGFR1 KD are coloured in blue and red, respectively. The gatekeeper residue V561 is shown and labelled in the structures. DFG-motif and the tyrosine residues 653, 654 are shown as ball-and-stick.

Supplemental Figure S4. Central cluster structure of active FGFR1^{V561M} based on MD simulations

The M561 is shown in yellow surrounded by the hydrophobic residues within 5Å shown with filled spheres and constituting the hydrophobic pocket. The residues that are considered part of the hydrophobic spine are shown in orange. The K514 and the E531 are shown in blue and red sticks and form a stable salt bridge. The activation loop is shown in pink. Residues that constitute molecular brake (N546, E562 and K638) are also indicated and the change in their position between the M561 variant (yellow) and V561 (WT) variant (grey) is shown in the inset.

Supplemental Figure S5. Characterization of NIH 3T3 cell lines stably expressing FGFR3 variants

A. Growth of the NIH 3T3 cell lines expressing FGFR3 WT, FGFR3 V555M, FGFR3TACC3 and FGFR3TACC3 V555M variants was monitored over 11 days.

B. Viability of the indicated cell lines was assessed by cell titre blue assay following treatment with increasing concentrations of PD173074 inhibitor and expressed relative to viability of untreated cells expressing FGFR3 WT (viability = 1).

Supplemental Figure S6. FGFR-inhibitors and their binding to FGFR1 KD

A. Chemical structures of FGFR-inhibitors: TKI258, AP24534, PD173074, AZD4547 and BGJ398

B. Comparison of inhibitor/ATP binding. In all panels the residues at the binding site are shown as ball-and-stick model and are coloured in cyan, light pink, limon, pink and purple for PDB codes 4UWZ (WT FGFR1 KD + TKI258), 2FGI (WT FGFR1 + PD173074), 3TT0 (WT FGFR1 + BGJ398), 3GQI (Activated FGFR1 + ACP) and 3OXZ (Abl + AP24534), respectively. The fluoride atom in TKI258 is coloured in forest green, the phosphate atoms in ACP is coloured in orange, nitrogen atoms in blue and oxygen atoms in red. The colours defined for each PDB code and ligands are for the carbon atoms.

Top panel: Comparison of binding pockets for PD173074 (forest green), BGJ398 (light orange) and TKI258 (chocolate)

Middle panel: Comparison of binding pockets for ACP (purple) and TKI258 (chocolate)

Bottom panel: Comparison of binding pockets for AP24534 (lime) and TKI258 (chocolate)

Supplemental Figure S7. Comparison of binding of the novel FGFR inhibitor FIIN-2 with the binding of TKI258

A. Chemical structures of TKI258 and FIIN-2.

B. The novel irreversible inhibitor FIIN-2 (pink) bound to FGFR4 gatekeeper mutant V550L (PDB: 4QQ5) is shown superposed onto FGFR1 gatekeeper mutant V561M bound to TKI258 (chocolate).

Supplemental Figures

A

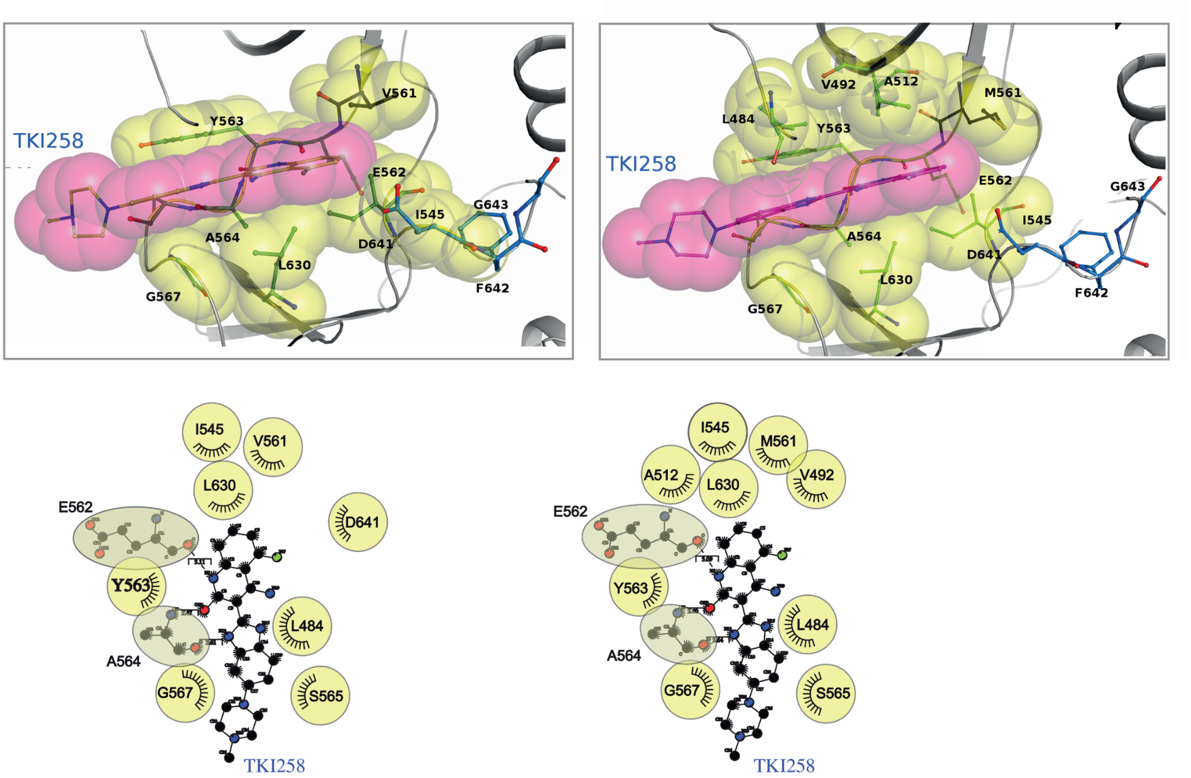

B

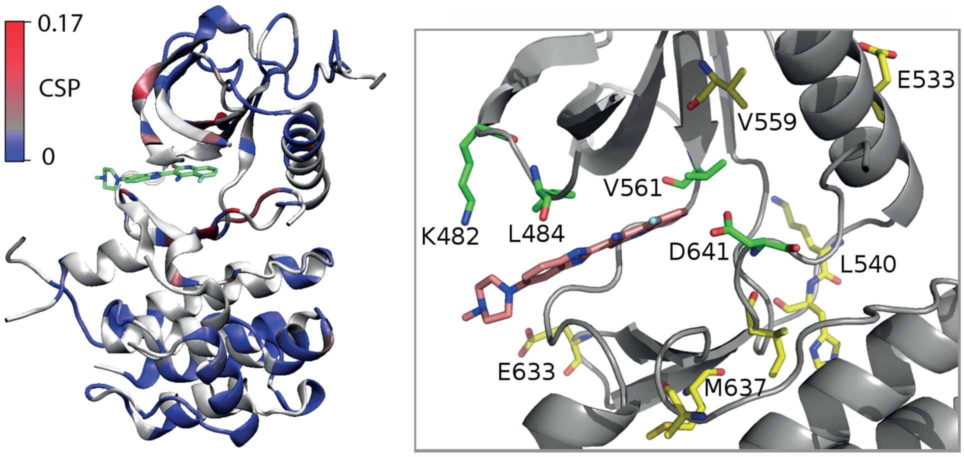

Supplemental Figure S1

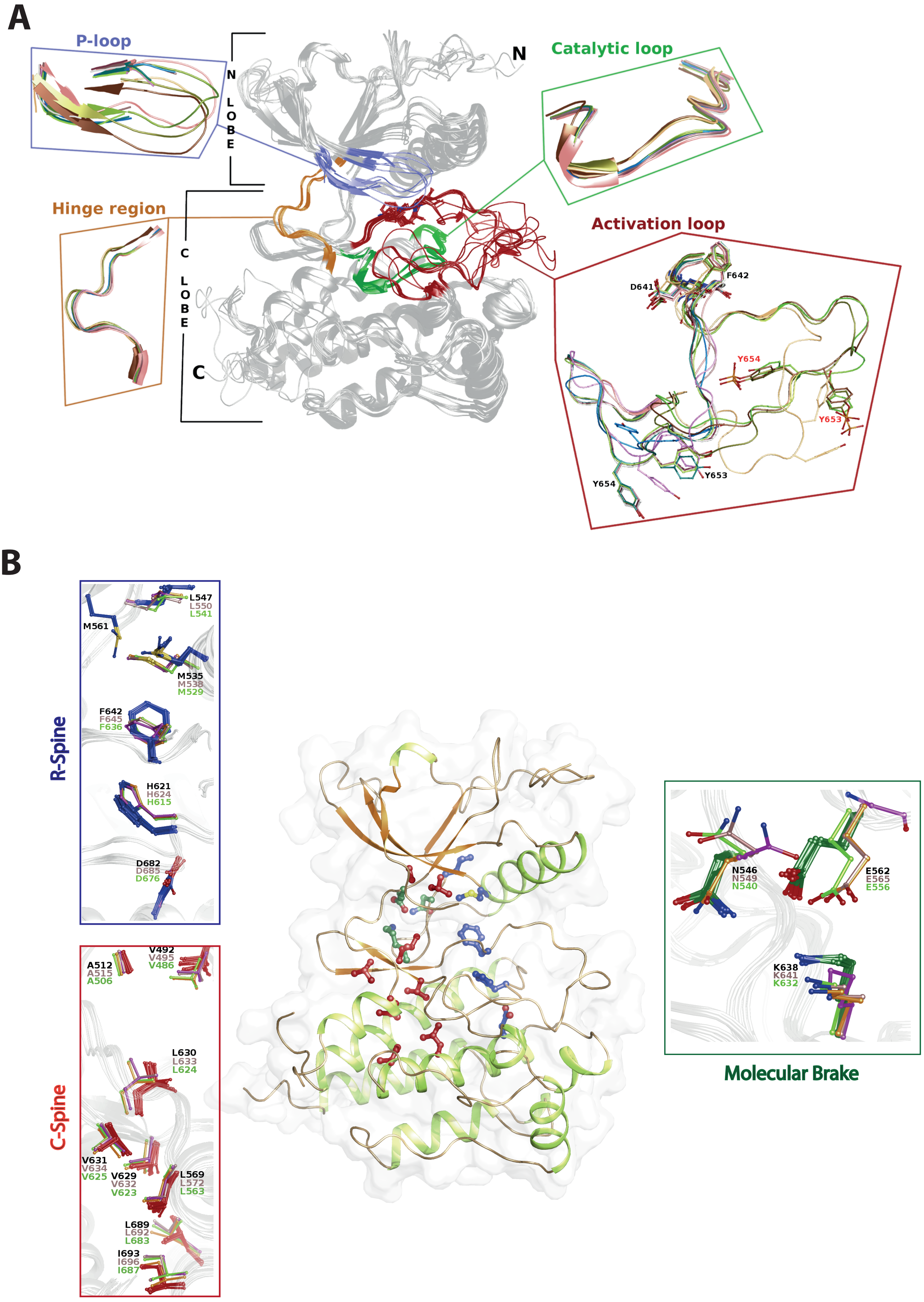

Supplementary Figure 2

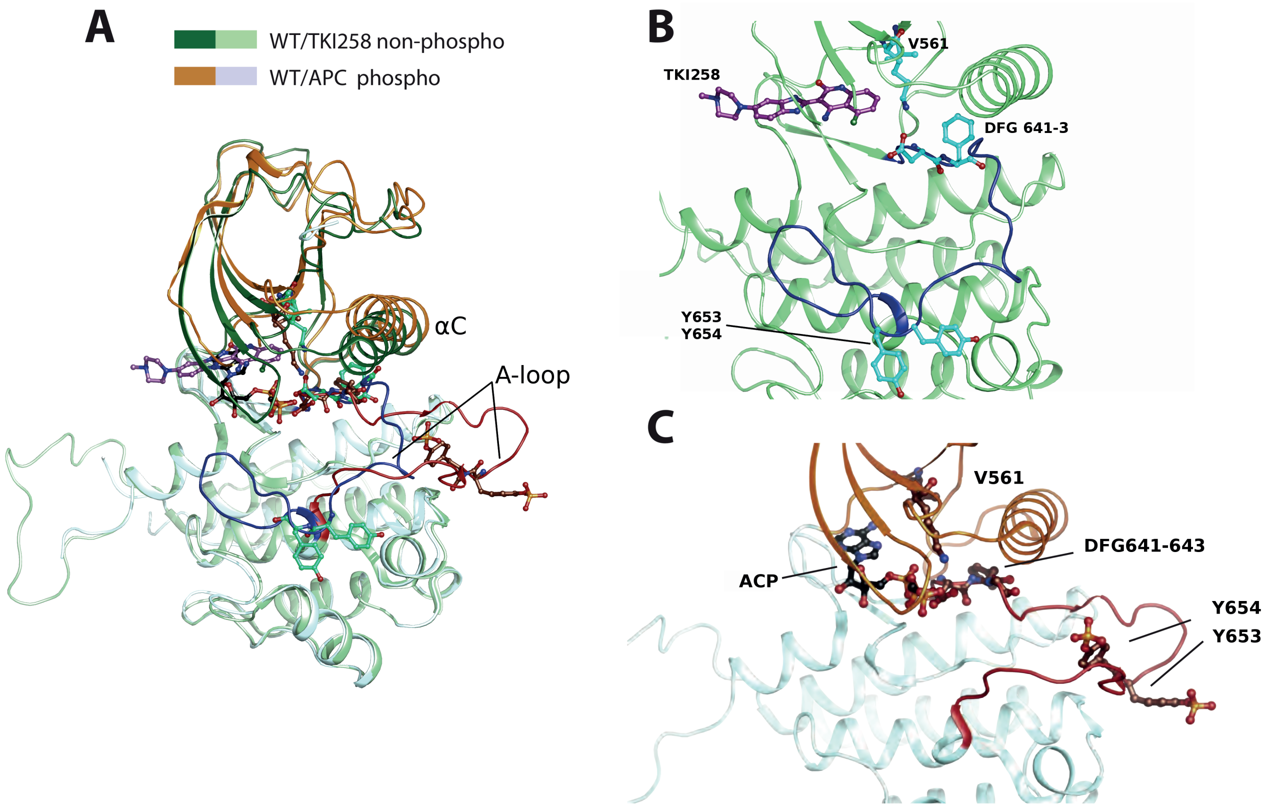

Supplemental Figure S3

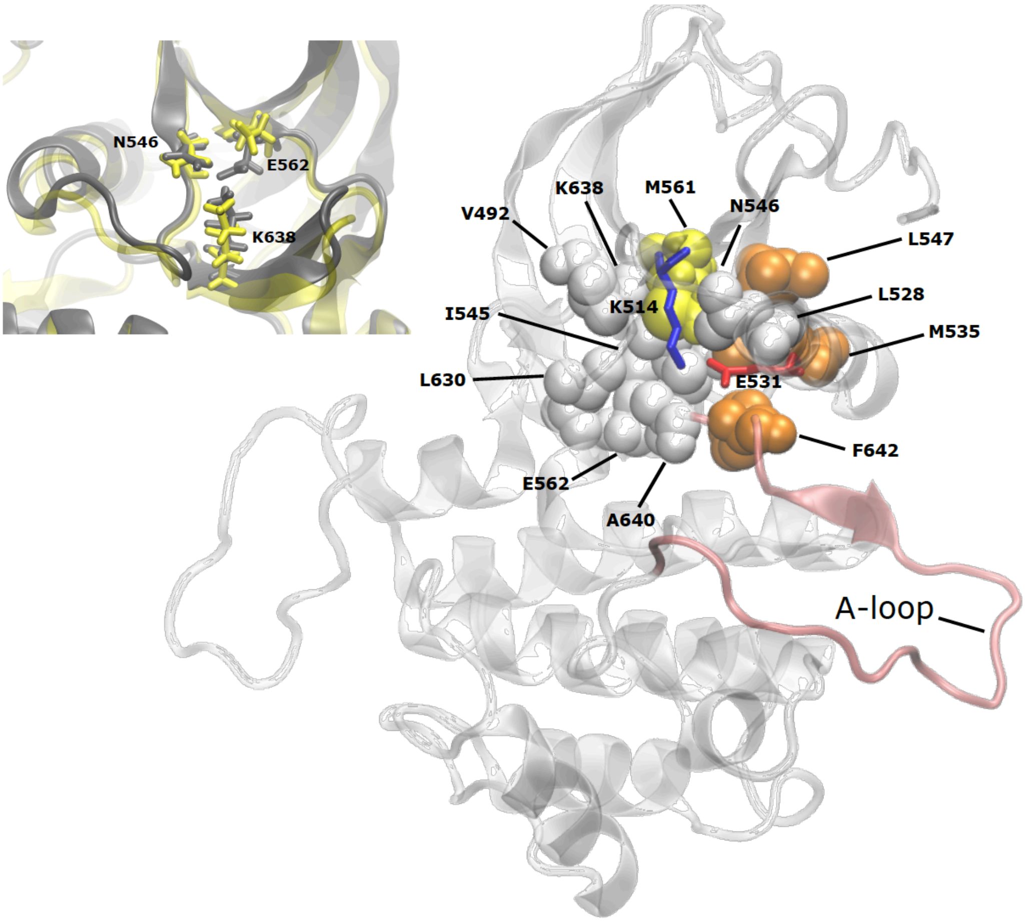

Supplemental Figure S4

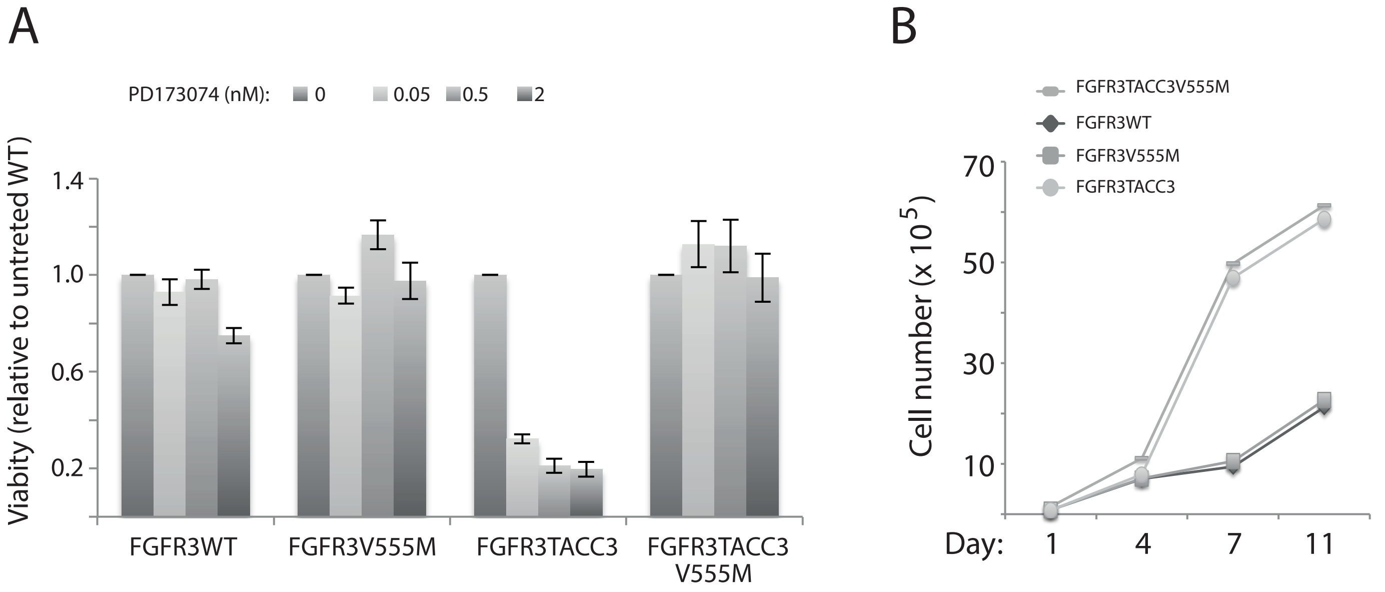

Supplmental Figure S5

TKI258

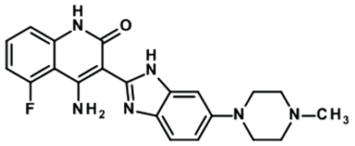

AP24534

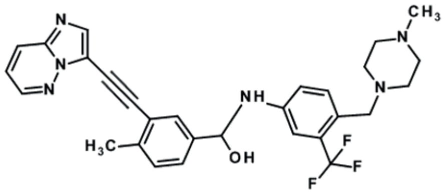

PD173074

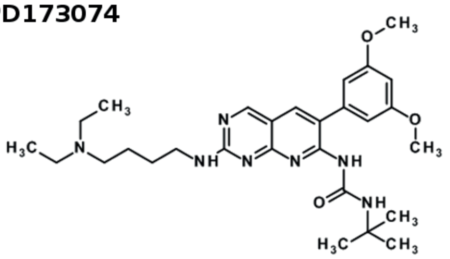

AZD4547

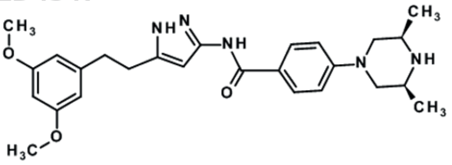

BGJ398

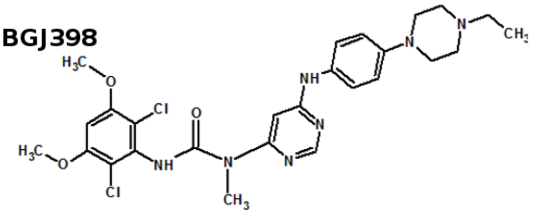

TKI258/BGJ398/PD173074

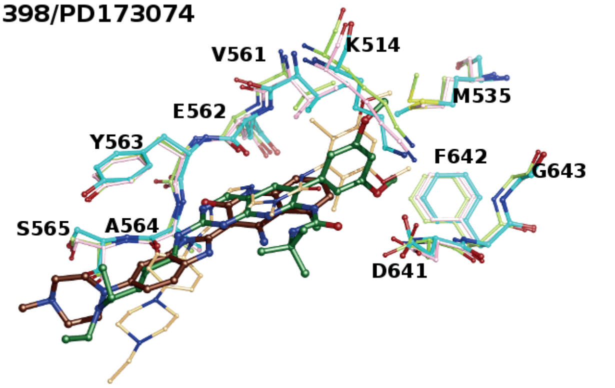

TKI258/ACP

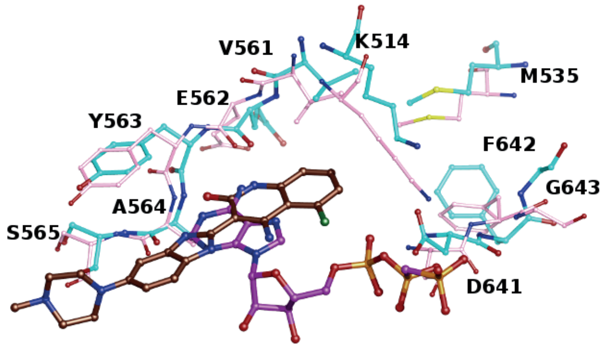

TKI258/AP24534

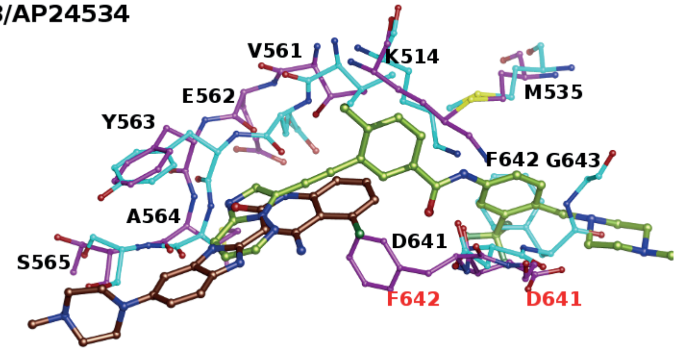

Supplemental Figure S6

A

TKI258

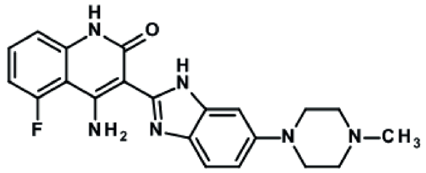

FIIN-2

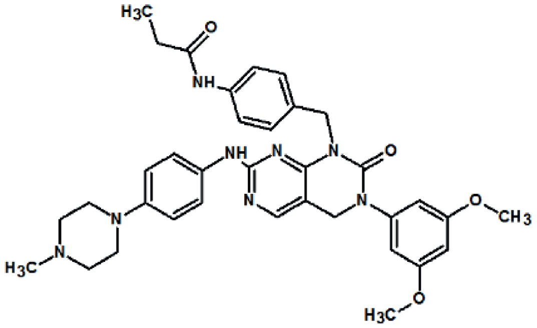

B

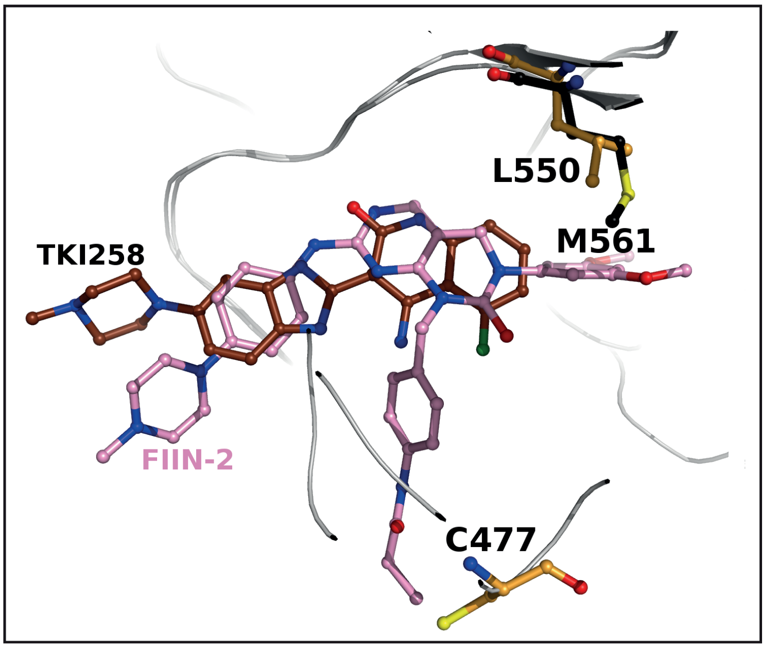

Supplmental Figure S7
